# Association of functional outcomes between intravenous tirofiban and endovascular thrombectomy in imaging-screened patients with large vessel occlusion stroke: a secondary analysis of randomized clinical trial

**DOI:** 10.1097/JS9.0000000000001666

**Published:** 2024-05-24

**Authors:** Li Wang, Jiacheng Huang, Jiaxing Song, Jie Yang, Linyu Li, Changwei Guo, Qingwu Yang, Wenjie Zi, Fengli Li, Weilin Kong

**Affiliations:** a Department of Neurology, Zigong Third People’s Hospital, Zigong, People’s Republic of China; b Department of Neurology, Xinqiao Hospital and The Second Affiliated Hospital, Army Medical University (Third Military Medical University), Chongqing, People’s Republic of China; c Department of Neurosurgery, General Hospital of Southern Theatre Command, Guangzhou, People's Republic of China

**Keywords:** endovascular thrombectomy, functional outcomes, large vessel occlusion stroke, optimizing patient selection, tirofiban

## Abstract

**Background:**

In the RESCUE BT (endovascular treatment with versus without tirofiban for stroke patients with large vessel occlusion) trial, enrollment in extended time window was based on noncontrast computed tomography. To assess whether perioperative intravenous tirofiban would further enhance the clinical benefit of endovascular therapy in the RESCUE BT trial according to advanced imaging criteria based on current American Heart Association/American Stroke Association (AHA/ASA) guidelines.

**Methods:**

This is a secondary analysis of the RESCUE BT trial. Patients who were eligible for endovascular thrombectomy in the 6 h window and met the criteria of the DAWN or DEFUSE 3 trials in the extended window according to the AHA/ASA guidelines were analyzed. The primary outcome was the distribution of the 90-day modified Rankin Scale (mRS) scores. Safety outcomes included the incidence of symptomatic intracranial hemorrhage (sICH) within 48 h and 90-day mortality.

**Results:**

A total of 652 patients (319 in tirofiban group and 333 in placebo group) who meeting the AHA/ASA guidelines were included in this analysis, with median interquartile ranges (IQR) age of 68 (58–75) years, 278 (42.6%) were women. The median 90-day mRS score was 3 (IQR, 1–4) in the tirofiban group, and 3 (IQR, 1–4) in the placebo group. The adjusted common odds ratio (OR) for a lower level of disability with tirofiban than with placebo was 1.08 (95% CI: 0.83–1.42). The incidence of sICH [10.1% versus 6.3%; adjusted OR 1.70; (95% CI: 0.95–3.04)] was not significantly different between groups. However, intravenous tirofiban might be associated with lower disability level [adjusted common OR, 1.74 (95% CI: 1.14–2.65); *P*=0.01] in patients with large artery atherosclerosis.

**Conclusions:**

There was no significant difference in the severity of disability at 90 days with intravenous tirofiban compared to placebo in patients who underwent endovascular therapy according to AHA/ASA guidelines. The authors observed potential benefits of tirofiban in patients with large artery atherosclerosis, but there was an increased risk of sICH in patients with cardioembolism stroke.

## Introduction

Highlights

- Intravenous tirofiban did not significantly improve the level 90-day global disability among patients with acute ischemic stroke due to anterior circulation large vessel occlusion in patients who met the recent American Heart Association guidelines.
- Intravenous tirofiban had potential benefits for improved functional outcome in the large artery atherosclerosis subgroup.
- Intravenous tirofiban increased the incidence of intracranial hemorrhage, especially significantly increased the incidence of symptomatic intracranial hemorrhage in patients with cardioembolism stroke, it seemed to lead to higher odds of death.

Endovascular therapy (EVT) following intravenous thrombolysis has become the current standard treatment for acute ischemic stroke (AIS) patients with a large vessel occlusion (LVO) in the anterior circulation^1^, as it has been robustly associated with significantly improved functional outcomes and prolonged survival^2^. However, although the proportion of patients with successful recanalization can be more than 90%, the proportion of patients with favorable outcome is still less than 50%, that is, nearly half of patients with successful recanalization belong to futile recanalization^3^. A large number of previous studies have shown that mechanical injury during the operative procedures, which causes endothelial damage to the arterial wall and aggravates platelet aggregation^4,5^, and microcirculatory dysfunction may be important factors restricting the clinical benefit of successful recanalization in patients^6^, except for age, severity of stroke, and other irresistible factors^7^. Therefore, simply increasing the proportion of recanalization does not radically improve the net clinical benefit, and a comprehensive strategy of perioperative administration of antiplatelet or anticoagulant therapy may be a potential therapeutic modality to reduce futile recanalization after EVT.

Unfortunately, the Multicenter Randomized Clinical Trial of Endovascular Treatment for Acute Ischemic Stroke in the Netherlands (MR CLEAN-MED) trial showed that periprocedural intravenous aspirin and unfractionated heparin during EVT did not increase the beneficial effect on functional outcome, but instead increased the risk of symptomatic intracranial hemorrhage^8^. Similarly, the Endovascular Treatment With versus Without Tirofiban for Stroke Patients with Large Vessel Occlusion (RESCUE BT) trial suggested that perioperative tirofiban, a highly selective nonpeptide glycoprotein IIb/IIIa receptor antagonist, did not improve the disability severity at 90 days in AIS patients undergoing EVT^9^. The RESCUE BT trial included some patients with an extended time window (6–24 h) and did not fully follow the current American Heart Association/American Stroke Association (AHA/ASA) guidelines for advanced imaging screening of patients in extended time window^10^. Given the heterogeneity of clinical and imaging characteristics in the early time window (0–6 h) and extended time window, as well as the different imaging modalities, the selection criteria of patients with EVT in different time windows. Recent comparisons of basic imaging and advanced imaging for evaluation of AIS in the extended time window have not shown significant differences in clinical outcomes between baseline imaging modalities^11,12^. However, it remains unclear whether screening with advanced imaging improves the clinical outcomes of perioperative intravenous tirofiban during EVT.

In this secondary analysis of the RESCUE BT trial, we aimed to comprehensively evaluate the safety and efficacy of intravenous tirofiban in combination with EVT in patients with AIS by optimizing patient selection with advanced imaging profiles according to recent AHA/ASA guidelines and according to the DAWN or DEFUSE-3 trial criteria, and to determine whether perioperative intravenous tirofiban would further enhance the clinical benefit of EVT.

## Methods

### Study design

The RESCUE BT trial was a multicenter, double-blinded, randomized, placebo-controlled trial conducted at 55 centers in China and enrolled 950 participants from 10 October 2018 to 31 October 2021. The study was approved by the ethics committees of the Xinqiao Hospital, Army Medical University, and all participating centers prior to the start of the study, and all enrolled patients or their legally authorized representatives provided written informed consent before enrollment. This secondary analysis of the RESCUE BT data followed the Consolidated Standards of Reporting Trials (CONSORT, Supplemental Digital Content 1, http://links.lww.com/JS9/C662, Supplemental Digital Content 2, http://links.lww.com/JS9/C663) reporting guideline^13^.

The design^14^ and primary results of the RESCUE BT trial have been published previously^9^. Briefly, consecutive AIS patients with anterior circulation LVO of the intracranial internal carotid artery, or the M1-segments or M2-segments of the middle cerebral artery who were eligible for EVT within 24 h of stroke onset after last known well. All patients underwent a unified prespecified imaging protocol with noncontrast computed tomography (NCCT), CT angiography (CTA), and CT perfusion (CTP) or MRI with core infarct and mismatch determination using the Fast-Processing of Ischemic Stroke software (Neuroblem Ltd. Company). All images were evaluated by an independent neuroimaging core laboratory (headed by Z. WJ) at the Xinqiao Hospital of Army Medical University, blinded to clinical outcome and enrollment site. Modified Rankin Scale (mRS) score assessment was performed at 90 days by neurologists blinded to the core laboratory reading and treatment assignment (tirofiban versus placebo).

### Study population

The time window was defined as the last known well to randomization. If patients presented in an early time window, the last simple imaging with NCCT of the head was the primary imaging selection modality prior to EVT. If NCCT was not available or the time window extended beyond 6 h and up to 24 h, patients were referred to advanced imaging, including CTP or MRI, to assess final infarct size with reference to the DAWN and DEFUSE-3 inclusion criteria^15,16^ and image analysis in Supplementary Methods (Supplemental Digital Content 3, http://links.lww.com/JS9/C664). If both NCCT and CTP were available, the patients were classified as selected by CTP. If both CTP and MRI were available, the patients were classified as selected by MRI. We excluded patients who did not receive CTP or MRI within an extended time window according to the DAWN/DEFUSE-3 criteria, based on current AHA/ASA guidelines, in order to better analyze and evaluate the safety and efficacy of perioperative intravenous tirofiban in AIS-LVO patients more accurately. These criteria reflect the broader range of inclusion of patients included in both the DAWN and DEFUSE 3 trials^15,16^.

### Study interventions

All patients who received EVT and the intravenous drug (tirofiban or placebo) were included in this secondary analysis, both in the early and extended time windows. EVT primarily included the use of stent retrievers, aspiration or other thrombectomy devices according to the recent AHA/ASA guidelines^10^. According to the randomization sequence, the study drug was administrated intravenously as a bolus dose of 10 μg/kg within 5 min after randomization, followed by continuous infusion of 0.15 μg/kg/min for up to 24 h. Details of the interventions have been published previously^14^.

### Study outcomes

The primary efficacy outcome was the functional improvement at 90 days, defined as a 1-point decrease across all mRS score (ordinal shift analysis). The secondary efficacy outcome included excellent outcome (mRS score, 0–1), functionally independent (mRS score, 0–2), and favorable outcome (mRS score, 0–3) at 90 days^17^. Safety outcomes included the incidence of symptomatic intracerebral hemorrhage (sICH) according to the Heidelberg classification with 48 h^18^, and mortality within 90 days. Imaging outcomes for patients treated with EVT were the postprocedure expanded treatment in cerebral ischemia (eTICI) score (with a successful reperfusion defined by a score ≥2b)^19^. The full list of outcomes has been published previously in this trial protocol^14^.

### Statistical analysis

For the continuous variables, we tested the normal distribution of the data with the Shapiro–Wilk test. As the normality tests showed significant results, the data were considered non-normal distribution, and hence, continuous data were presented as medians with corresponding interquartile ranges (IQR). Otherwise, the data were considered to be normally distributed continuous variables, and are presented as means with corresponding SD. Nonparametric Mann–Whitney *U* tests or 2-sample *t*-test were used to examine differences in continuous variables. For categorical variables, the data were reported using their as absolute numbers and percentages and *χ* ^2^ tests (or Fisher exact tests) were used to assess for differences.

For the distribution of 90-day mRS scores, a multivariable ordinal logistic regression model was performed to estimate a 1-point shift towards the lower ordered value, indicating a better outcome. The adjusted common odds ratios (ORs) of shifting from one category to the next were reported with 95% CIs. For the outcome of favorable outcomes at 90 days, a multivariable binary logistic regression model was used to compare the odds of a favorable outcome in the tirofiban and placebo groups. Clinically important variables [e.g. age, baseline National Institutes of Health Stroke Scale (NIHSS) score, baseline Alberta Stroke Program Early CT Score (ASPECTS), occlusion site, and time from onset to randomization] were entered into the model. The adjusted ORs with 95% CIs for functional independence were reported.

To determine whether tirofiban could mediate the effect of increased sICH on the risk of death after EVT, we performed a mediation analysis to calculate the proportion mediated and to test its significance. A mediation analysis consists of a four-step procedure and attempts to explain a relationship between an independent variable (tirofiban) and a dependent variable (death) via the inclusion of a third hypothetical variable (sICH). All analyses were performed using R version 4.1.0 software and SPSS statistical software (version 26.0, IBM), and two-side *P*-value with significance threshold of *P*<0.05 were considered statistically significant. Data were analyzed from April 2023 to June 2023.

## Results

### Baseline characteristics

**Figure 1:**
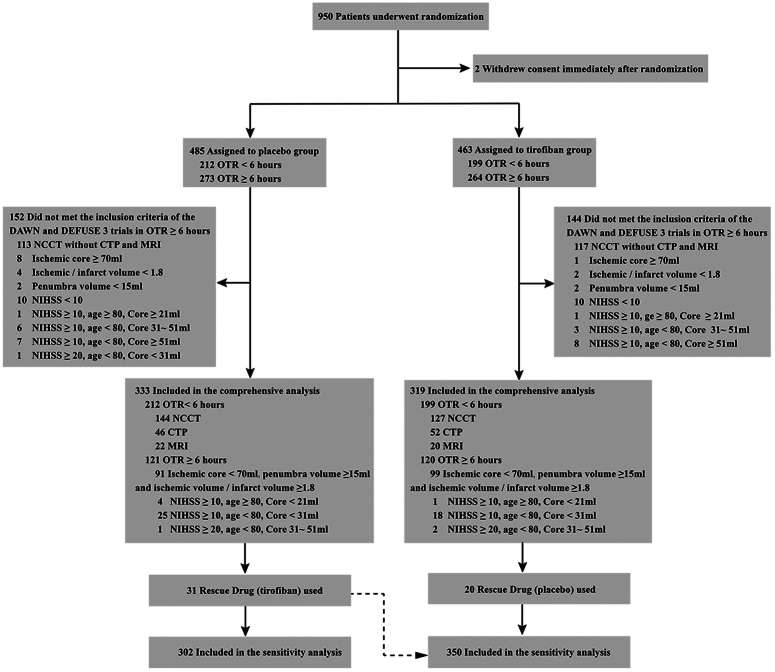
Study flow diagram. OTR, onset to randomization; DAWN, DWI, or CTP, assessment with clinical mismatch in the triage of wake-up and late presenting strokes undergoing neurointervention with trevo; DEFUSE, endovascular therapy following imaging evaluation for ischemic stroke; NCCT, noncontrast computed tomography; CTP, computed tomography perfusion; NIHSS, National Institutes of Health Stroke Scale.

The study profile of patients screened, enrolled, and analyzed is presented in the Figure 1. A total of 950 patients underwent randomization in the RESCUE BT trial, with two patients withdrawing consent immediately after randomization and receiving neither study drug nor EVT. Of these, 296 patients were considered ineligible due to lack of advanced imaging data in the extended time window and 652 patients met the current guidelines and entered the secondary analysis, of whom 319 were assigned to the tirofiban group (199 in the early time window and 120 in the extended time window) and 333 to the placebo group (212 in the early time window and 121 in the extended time window). Thirty-one patients who received tirofiban therapy in the placebo group were included in the tirofiban group in the sensitivity analysis. The demographics, medical history, baseline, imaging characteristics, and procedural characteristics of patients included and excluded from the RESCUE BT trial were described and compared in Table 1 and Supplementary Material Table S1, S2 (Supplemental Digital Content 3, http://links.lww.com/JS9/C664).

**Table 1:** Baseline characteristics according to treatment group in participants with large vessel occlusion stroke.

Characteristic	Overall (*n*=652)	Placebo group (*n*=333)	Tirofiban group (*n*=319)	*P*
Demographic characteristics				
Age, median (IQR)	68 (58–75)	68 (58–75)	68 (58–74)	0.70
Sex, no. (%)				0.99
Women	278 (42.6)	142 (42.6)	136 (42.6)	
Men	374 (57.4)	191 (57.4)	183 (57.4)	
Medical history, no. (%)^a^				
Hypertension	371 (56.9)	190 (57.1)	181 (56.7)	0.94
Diabetes	152 (23.3)	74 (22.2)	78 (24.5)	0.50
Hyperlipidemia	92 (14.1)	44 (13.2)	48 (15.0)	0.44
Atrial fibrillation	232 (35.6)	114 (34.2)	118 (37.0)	0.46
Ischemic stroke	112 (17.2)	57 (17.1)	55 (17.2)	0.97
Smoking (Current or past)	147 (22.5)	79 (23.7)	68 (21.3)	0.46
Prestroke modified Rankin scale score				0.30
0	592 (90.8)	299 (89.8)	293 (91.8)	
1	42 (6.4)	26 (7.8)	16 (5.0)	
2	16 (2.5)	7 (2.1)	9 (2.8)	
3	1 (0.2)	1 (0.3)	0 (0.0)	
4	1 (0.2)	0 (0.0)	1 (0.3)	
Stroke etiology, no. (%)				0.18
Large artery atherosclerosis	287 (44.0)	156 (46.8)	131 (41.1)	
Cardioembolism	299 (45.9)	151 (45.3)	148 (46.4)	
Unknown	48 (7.4)	19 (5.7)	29 (9.1)	
Other	18 (2.8)	7 (2.1)	11 (3.4)	
Clinical characteristics				
Baseline NIHSS score, median (IQR)	16 (12–19)	16 (12–19)	16 (12–19)	0.64
Baseline systolic blood pressure, median (IQR), mmHg	145 (130–160)	145 (130–160)	146 (130–160)	0.58
Baseline diastolic blood pressure, median (IQR), mmHg	84 (76–94)	84 (76–95)	83 (75–94)	0.67
Baseline serum glucose, median (IQR), mmol/l^b^	6.9 (5.8–8.7)	6.9 (5.8–8.7)	6.9 (5.7–8.6)	0.55
Imaging characteristics, no. (%)				0.25
NCCT	271 (41.6)	144 (43.2)	127 (39.8)	
CTP	288 (44.2)	137 (41.1)	151 (47.3)	
MRI	93 (14.3)	52 (15.6)	41 (12.9)	
Baseline ASPECTS, median (IQR)	8 (7–9)	8 (7–9)	8 (7–9)	0.63
Occlusion site				0.52
ICA intracranial	130 (19.9)	67 (20.1)	63 (19.7)	
Middle cerebral artery				
M1 segment	424 (65.0)	211 (63.4)	213 (66.8)	
M2 segment	98 (15.0)	55 (16.5)	43 (13.5)	
Collateral status, no. (%)^c^				0.64
ASITN/SIR grade 0	54 (8.3)	31 (9.3)	23 (7.2)	
ASITN/SIR grade 1	151 (23.2)	80 (24.0)	71 (22.3)	
ASITN/SIR grade 2	261 (40.1)	133 (39.9)	128 (40.3)	
ASITN/SIR grade 3	179 (27.5)	85 (25.5)	94 (29.6)	
ASITN/SIR grade 4	6 (0.9)	4 (1.2)	2 (0.6)	
Tandem lesion, no. (%)				0.96
No	617 (94.6)	316 (94.9)	301 (94.4)	
Severe stenosis of extracranial segment (≥70%)	25 (3.8)	12 (3.6)	13 (4.1)	
Extracranial occlusion	10 (1.5)	5 (1.5)	5 (1.6)	
Total passes, median (IQR)	2 (1–2)	2 (1–2)	1 (1–2)	0.10
First pass effect, no. (%)	178 (27.3)	82 (24.6)	96 (30.1)	0.12
Stent thrombectomy only, no. (%)	93 (14.3)	52 (15.6)	41 (12.9)	0.31
Aspiration only, no. (%)	126 (19.3)	60 (18.0)	66 (20.7)	0.39
Salvage therapy, no. (%)	129 (19.8)	74 (22.2)	55 (17.2)	0.11
SWIM only, no. (%)	119 (18.3)	55 (16.5)	64 (20.1)	0.24
Anesthesia, no. (%)				0.93
General	226 (34.7)	116 (34.8)	110 (34.5)	
Local	426 (65.3)	217 (65.2)	209 (65.5)	
Onset to randomization, median (IQR), min	386 (283–619)	369 (275–601)	395 (289–629)	0.31
Door to puncture, median (IQR), min	105 (78–144)	100 (79–141)	106 (75–145)	0.29
Puncture to recanalization, median (IQR), min	70 (40–108)	70 (41–110)	70 (40–104)	0.64

^a^ Patient self-report or family report.

^b^ Serum glucose data was not available for 19 and 22 patients in placebo group and tirofiban group, respectively.

^c^ ASITN/SIR by DSA data was not available for 1 patient in tirofiban group.

ASITN/SIR, American Society of Intervention and Therapeutic Neuroradiology/Society of Interventional Radiology; ASPECTS, Alberta Stroke Program Early CT Score; CTP, computed tomography perfusion; EVT, endovascular therapy; ICA, internal carotid artery; IQR, interquartile range; NCCT, noncontrast computed tomography; NIHSS, National Institutes of Health Stroke Scale.

### Primary efficacy outcome

**Figure 2:**
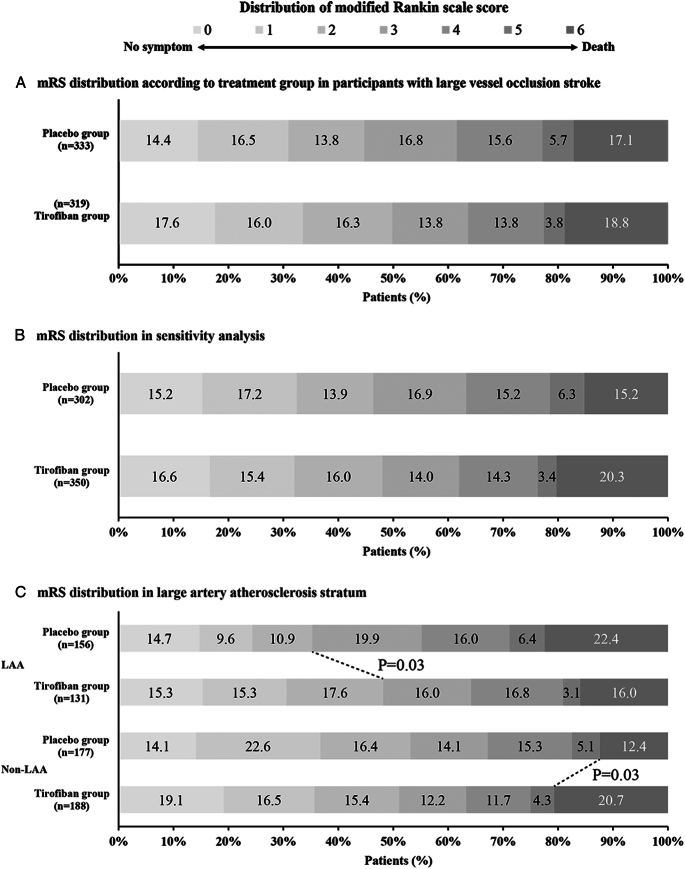
Distribution of Score on the Modified Rankin Scale (mRS) at 90 Days. This figure shown the 90-day mRS scores distribution between the tirofiban group and the placebo group. A, distribution of score on the mRS at 90 days in participants with large vessel occlusion stroke. B, distribution of score on the mRS at 90 days in sensitivity analysis with large vessel occlusion stroke. C, distribution of score on the mRS at 90 days stratified by stroke etiology. Bars are labeled with percentages. Scores on the mRS of functional disability range from 0 (no symptoms) to 6 (death).

**Figure 3:**
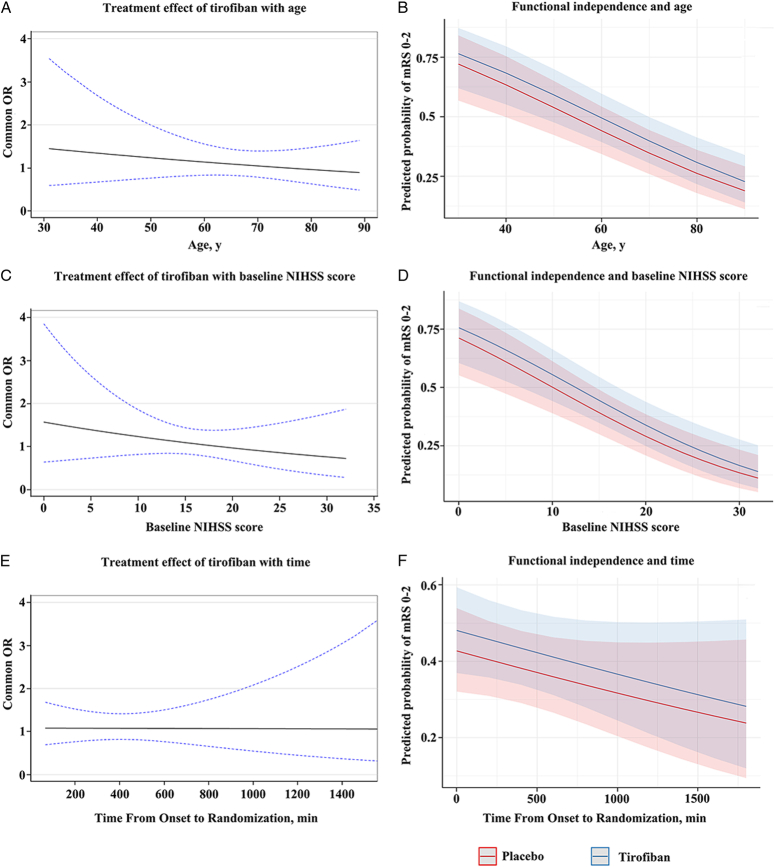
Tirofiban treatment effect and probability of functional independence. The three graphs on the left show the treatment effect of intravenous tirofiban, expressed as the common odds ratio (OR) for improved functional outcome (solid black lines) and its 95% CI (dashed blue lines) expressed as a function of age (A), baseline National Institutes of Health Stroke Scale (NIHSS) score (C), and time from onset to randomization (E). The treatment effect did not change with any of these baseline variables. The three graphs on the right show the probability and 95% CI of functional independence at 90 days, separately for patients in the placebo arm (light red lines and shading) and the tirofiban treatment arm (light blue lines and shading). The probability of functional independence was not associated with age (B), baseline NIHSS score (D) and time from onset to randomization (F).

The median (IQR) 90-day mRS score was 3 (1–4) in the tirofiban group and 3 (1–4) in the placebo group. In the multivariable analysis, after adjustment for confounder factors, there was no difference between tirofiban or placebo for a favorable shift to a lower 90-day mRS score [adjusted common ORs, 1.08 (95% CIs: 0.83–1.42); *P*=0.57; Table 2; Figure 2A and Supplementary Material Table S3, Supplemental Digital Content 3, http://links.lww.com/JS9/C664]. There was no interaction between tirofiban and age (adjusted common ORs for improved functional outcome with each year of older age, 0.99; 95% CIs: 0.97–1.02; *P* for interaction=0.55; range 31–89 years), baseline stroke severity (adjusted common ORs for improved functional outcome with each point of higher NIHSS score, 0.98; 95% CIs: 0.92–1.03; *P* for interaction=0.40; range 0–32 score), or time from onset to randomization (adjusted common ORs for improved functional outcome with each hour of longer time to randomization, 1.00; 95% CIs: 0.94–1.06; *P* for interaction=0.96; range 1–24 h) (Fig. 3). At the same time, heterogeneity in the treatment effect of tirofiban was not observed in the AHA/ASA guideline-eligible and guideline-ineligible populations (Supplementary Material Figure S1, Supplemental Digital Content 3, http://links.lww.com/JS9/C664).

**Table 2:** Primary and secondary efficacy outcomes and safety outcomes according to treatment group in participants with large vessel occlusion stroke.

Characteristic	Placebo group (*n*=333)	Tirofiban group (*n*=319)	Unadjusted difference (95% CIs)	Unadjusted OR (95% CIs)	Adjusted OR (95% CIs)^a^	*P*
Primary efficacy outcome						
mRS score at 90 days, median (IQR)	3 (1–4)	3 (1–4)	0 (0 to 0)	1.12 (0.86–1.47)	1.08 (0.83–1.42)	0.57
Secondary efficacy outcomes						
Excellent outcomes at 90 days (%)	106 (31.8)	111 (34.8)	0.03 (−0.04–0.10)	1.14 (0.83–1.58)	1.13 (0.80–1.60)	0.50
Independent outcomes at 90 days (%)	149 (44.7)	159 (49.8)	0.05 (−0.03 to 0.13)	1.23 (0.90–1.67)	1.24 (0.89–1.72)	0.21
Favorable outcomes at 90 days (%)	205 (61.6)	203 (63.6)	0.02 (−0.06 to 0.09)	1.08 (0.79–1.49)	1.08 (0.76–1.52)	0.67
NIHSS change from baseline to 24 h, median (IQR)^b^	−2 (−6 to 2)	−2 (−7 to 2)	0 (−1 to 1)	0.39 (−1.02–1.79)	0.30 (−1.09–1.68)	0.67
NIHSS change from baseline to 5–7 days or early discharge, median (IQR)^b^	−5 (−11 to 1)	−5 (−10 to 0)	0 (−1 to 1)	0.33 (−1.48–2.15)	0.24 (−1.54–2.01)	0.79
EQ-5D-5L score at 90 d, median (IQR)^b^	0.6 (0.2–1.0)	0.7 (0.2–1.0)	0 (0 to 0.05)	0.02 (−0.04–0.08)	0.02 (−0.04–0.07)	0.56
Secondary technical efficacy outcomes						
Substantial reperfusion on initial DSA prior to EVT (%)	1 (0.3)	0 (0.0)	0.003 (−0.009 to 0.017)	NA	NA	NA
Substantial reperfusion at final angiogram EVT (%)	302 (90.7)	292 (91.5)	0.01 (−0.04–0.05)	1.11 (0.65–1.91)	1.11 (0.64–1.92)	0.71
Recanalization on follow-up CTA or MRA within 48 h (%)^c^	217 (87.1)	202 (87.4)	0.02 (−0.05 to 0.09)	1.03 (0.60–1.76)	1.01 (0.58–1.74)	0.99
Primary safety outcomes						
Symptomatic ICH within 48 hours (%)^d^	21 (6.3)	32 (10.1)	0.04 (−0.005 to 0.08)	1.66 (0.94–2.95)	1.70 (0.95–3.04)	0.08
Any radiologic ICH within 48 h (%)^e^	93 (27.9)	113 (35.5)	0.07 (0.003–0.145)	1.42 (1.02–1.98)	1.45 (1.03–2.04)	0.03
Death at 90 days (%)	57 (17.1)	60 (18.8)	0.02 (−0.04–0.08)	1.12 (0.75-1.67)	1.17 (0.77-1.78)	0.46

^a^ Values were adjusted for age, baseline NIHSS score, baseline ASPECTS, occlusion site, and time from last known well to randomization, as prespecified in the protocol and statistical analysis plan.

^b^ The β coefficient was estimated from a linear regression model.

^c^ Recanalization on follow-up CTA or MRA within 48 h data was not available for 84 and 88 patients in placebo group and tirofiban group, respectively.

^d^ Symptomatic ICH within 48 h data was not available for 1 patient in tirofiban group.

^e^ Any radiologic ICH within 48 h data was not available for 1 patient in tirofiban group.

CTA, computed tomography angiography; DSA, digital subtraction angiography; European Quality Five-Dimension Five-Level Self-Report Questionnaire (EQ-5D-5L); EVT, endovascular therapy; ICH, intracerebral hemorrhage; IQR, interquartile range; MRA, magnetic resonance angiography; NA, not applicable; NIHSS, National Institutes of Health Stroke Scale.

### Secondary efficacy outcomes

Likewise, the crude and adjusted ORs of secondary outcomes were presented in Table 2. There was no statistically significant difference in 90-day excellent outcome [adjusted ORs, 1.13 (95% CIs: 0.80–1.60); *P*=0.50], functional independence [adjusted ORs, 1.24 (95% CIs: 0.89–1.72); *P*=0.21] and favorable outcome [adjusted ORs, 1.08 (95% CIs: 0.76–1.52); *P*=0.67] between the tirofiban group and placebo group. The probability of being functionally independent at 90 days declined with older age and higher NIHSS score between in the tirofiban and placebo groups (Fig. 3). The technical efficacy outcomes did not differ between patients receiving tirofiban and those not receiving tirofiban, with rates of successful reperfusion at the final angiogram [eTICI ≥2b, tirofiban 292 (91.5%) versus placebo 302 (90.7%), adjusted ORs 1.11, 95% CI: 0.64–1.92, *P*=0.71] and successful reperfusion achieved with first pass of stent-retriever [tirofiban 96 (30.1%) versus placebo 82 (24.6%), adjusted ORs 1.34, 95% CI: 0.94–1.90, *P*=0.11] were similar on both groups.

### Safety outcomes

Safety outcomes were shown in Table 2 and Supplementary Material Figure S2 (Supplemental Digital Content 3, http://links.lww.com/JS9/C664). There was no statistically significant difference in 90-day mortality between in the tirofiban group and placebo groups. Similarly, the incidence of sICH within 48 h was present in the placebo group [21 (6.3%)] and in the tirofiban group [32 (10.1%)]. However, the incidence of any radiologic ICH within 48 h was significantly higher in the tirofiban group than the placebo group [113 (35.5%) versus 93 (27.9%), adjusted ORs 1.45, 95% CI: 1.03–2.04]. The probability of 90-day mortality increased with older age, longer time from onset to randomization and higher NIHSS score between in the tirofiban and placebo groups, but declined with higher ASPECTS score. A similar pattern of changing probabilities were observed for sICH within 48 h in both tirofiban and placebo groups (Supplementary Material Figure S3, Supplemental Digital Content 3, http://links.lww.com/JS9/C664 and S4, Supplemental Digital Content 3, http://links.lww.com/JS9/C664). In addition, there were no statistically significant differences in adverse events and complications between the tirofiban and placebo groups (Supplementary Material Table S4, Supplemental Digital Content 3, http://links.lww.com/JS9/C664).

### Sensitivity analysis

In a sensitivity analysis, there were no baseline differences between the tirofiban and placebo groups (Table 3). There remained a nonsignificant statistical difference in ordinal mRS shifts between patients received tirofiban and placebo (Fig. 2B). Similarly, the odds of secondary efficacy outcomes and safety outcomes, adverse events and complications were similar on multivariate analysis between patients received tirofiban and placebo (Table 4 and Supplementary Material Table S5–7, Supplemental Digital Content 3, http://links.lww.com/JS9/C664).

**Table 3:** Baseline characteristics and workflow measures of the patients and features in sensitivity analysis.

Characteristic	Overall (*n*=652)	Placebo group (*n*=302)	Tirofiban group (*n*=350)	*P*
Demographic characteristics				
Age, median (IQR)	68 (58–75)	69 (59–76)	67 (58–74)	0.11
Sex, no. (%)				0.41
Women	278 (42.6)	134 (44.4)	144 (41.1)	
Men	374 (57.4)	168 (55.6)	206 (58.9)	
Medical history, no. (%)^a^				
Hypertension	371 (56.9)	167 (55.3)	204 (58.3)	0.44
Diabetes	152 (23.3)	66 (21.9)	86 (24.6)	0.41
Hyperlipidemia	92 (14.1)	40 (13.2)	52 (14.9)	0.56
Atrial fibrillation	232 (35.6)	111 (36.8)	121 (34.6)	0.56
Ischemic stroke	112 (17.2)	55 (18.2)	57 (16.3)	0.52
Smoking (Current or Past)	147 (22.5)	69 (22.8)	78 (22.3)	0.86
Prestroke modified Rankin scale score				0.21
0	592 (90.8)	269 (89.1)	323 (92.3)	
1	42 (6.4)	25 (8.3)	17 (4.9)	
2	16 (2.5)	7 (2.3)	9 (2.6)	
3	1 (0.2)	1 (0.3)	0 (0.0)	
4	1 (0.2)	0 (0.0)	1 (0.3)	
Stroke etiology, no. (%)				0.29
Large artery atherosclerosis	287 (44.0)	126 (41.7)	161 (46.0)	
Cardioembolism	299 (45.9)	150 (49.7)	149 (42.6)	
Unknown	48 (7.4)	19 (6.3)	29 (8.3)	
Other	18 (2.8)	7 (2.3)	11 (3.1)	
Clinical characteristics				
Baseline NIHSS score, median (IQR)	16 (12–19)	16 (12–20)	16 (12–19)	0.16
Baseline Systolic blood pressure, median (IQR), mmHg	145 (130–160)	144 (129–159)	146 (130–160)	0.42
Baseline Diastolic blood pressure, median (IQR), mmHg	84 (76–94)	84 (76–93)	83 (75–94)	0.97
Baseline Serum glucose, median (IQR), mmol/L^b^	6.9 (5.8–8.7)	6.9 (5.8–8.7)	6.9 (5.7–8.7)	0.80
Imaging characteristics, no. (%)				0.17
NCCT	271 (41.6)	137 (45.4)	134 (38.3)	
CTP	288 (44.2)	123 (40.7)	165 (47.1)	
MRI	93 (14.3)	42 (13.9)	51 (14.6)	
Baseline ASPECTS, median (IQR)	8 (7–9)	8 (7–9)	8 (7–9)	0.64
Occlusion site				0.16
ICA intracranial	130 (19.9)	63 (20.9)	67 (19.1)	
Middle cerebral artery				
M1 segment	424 (65.0)	186 (61.6)	238 (68.0)	
M2 segment	98 (15.0)	53 (17.5)	45 (12.9)	
Collateral status, no. (%)^c^				0.18
ASITN/SIR grade 0	54 (8.3)	31 (10.3)	23 (6.6)	
ASITN/SIR grade 1	151 (23.2)	76 (25.2)	75 (21.5)	
ASITN/SIR grade 2	261 (40.1)	115 (38.1)	146 (41.8)	
ASITN/SIR grade 3	179 (27.5)	76 (25.2)	103 (29.5)	
ASITN/SIR grade 4	6 (0.9)	4 (1.3)	2 (0.6)	
Tandem lesion, no. (%)				0.79
No	617 (94.6)	287 (95.0)	330 (94.3)	
Severe stenosis of extracranial segment (≥70%)	25 (3.8)	10 (3.3)	15 (4.3)	
Extracranial occlusion	10 (1.5)	5 (1.7)	5 (1.4)	
Total passes, median (IQR)	2 (1–2)	2 (1–2)	1 (1–2)	0.59
First pass effect, no. (%)	178 (27.3)	81 (26.8)	97 (27.7)	0.80
Stent thrombectomy only, no. (%)	93 (14.3)	50 (16.6)	43 (12.3)	0.12
Aspiration only, no. (%)	126 (19.3)	58 (19.2)	68 (19.4)	0.94
Salvage therapy, no. (%)	129 (19.8)	53 (17.5)	76 (21.7)	0.18
SWIM only, no. (%)	119 (18.3)	55 (18.2)	64 (18.3)	0.98
Anesthesia, no. (%)				0.96
General	226 (34.7)	105 (34.8)	121 (34.6)	
Local	426 (65.3)	197 (65.2)	229 (65.4)	
Onset to randomization, median (IQR), min	386 (283–619)	359 (270–590)	400 (292–631)	0.08
Door to puncture, median (IQR), min	105 (78–144)	99 (79–139)	110 (78–150)	0.04
Puncture to recanalization, median (IQR), min	70 (40–108)	66 (40–105)	72 (42–110)	0.54

^a^ Patient self-report or family report.

^b^ Serum glucose data was not available for 18 and 23 patients in placebo group and tirofiban group, respectively.

^c^ ASITN/SIR by DSA data was not available for 1 patient in tirofiban group.

ASITN/SIR, American Society of Intervention and Therapeutic Neuroradiology/Society of Interventional Radiology; ASPECTS, Alberta Stroke Program Early CT Score; CTP, computed tomography perfusion; ICA, internal carotid artery; IQR, interquartile range; NCCT, noncontrast computed tomography; NIHSS, National Institutes of Health Stroke Scale.

**Table 4:** Efficacy outcomes and primary safety outcomes in sensitivity analysis.

Characteristic	Placebo group (*n*=302)	Tirofiban group (*n*=3350)	Unadjusted difference (95% CI)	Unadjusted OR (95% CI)	Adjusted OR (95% CI)^a^	*P*
Primary efficacy outcome						
mRS score at 90 days, median (IQR)	3 (1–4)	3 (1–4)	0 (0 to 0)	0.97 (0.74–1.26)	0.88 (0.67–1.16)	0.84
Secondary efficacy outcomes						
Excellent outcomes at 90 days (%)	101 (33.4)	116 (33.1)	0.002 (−0.07 to 0.07)	0.99 (0.71–1.37)	0.89 (0.63–1.27)	0.52
Independent outcomes at 90 days (%)	140 (46.4)	168 (48.0)	0.02 (−0.06 to 0.09)	1.07 (0.79–1.45)	0.99 (0.71–1.38)	0.97
Favorable outcomes at 90 days (%)	191 (63.2)	217 (62.0)	0.01 (−0.06 to 0.08)	0.95 (0.69–1.30)	0.85 (0.60–1.20)	0.34
NIHSS change from baseline to 24 h, median (IQR)^b^	−2 (−6 to 2)	−2 (−7 to 2)	0 (−1 to 2)	0.64 (−0.77 to 2.05)	0.59 (−0.80–1.98)	0.41
NIHSS change from baseline to 5–7 days or early discharge, median (IQR)^b^	−5.5 (−11 to 1)	−4 (−10 to 0)	1 (−1 to 2)	1.16 (−0.67 to 2.98)	1.11 (−0.68 to 2.89)	0.22
EQ-5D-5L score at 90 d, median (IQR)^b^	0.7 (0.2–1.0)	0.7 (0.2–1.0)	0 (0 to 0)	−0.01 (−0.07 to 0.05)	−0.03 (−0.08 to 0.03)	0.33
Secondary technical efficacy outcomes						
Substantial reperfusion on initial DSA prior to EVT (%)	1 (0.3)	0 (0.0)	0.003 (−0.01 to 0.02)	NA	NA	NA
Substantial reperfusion at final angiogram EVT (%)	274 (90.7)	320 (91.4)	0.01 (−0.04–0.05)	1.09 (0.64–1.87)	1.07 (0.62–1.86)	0.80
Recanalization on follow-up CTA or MRA within 48 h (%)^c^	204 (91.1)	215 (84.0)	0.06 (−0.01 to 0.13)	0.51 (0.29–0.91)	0.54 (0.30–0.96)	0.04
Primary safety outcomes						
Symptomatic ICH within 48 h (%)^d^	18 (6.0)	35 (10.0)	0.04 (−0.002 to 0.08)	1.76 (0.97–3.18)	1.90 (1.04–3.48)	0.04
Any radiologic ICH within 48 h (%)^e^	87 (28.8)	119 (34.1)	0.05 (−0.02 to 0.12)	1.28 (0.92–1.78)	1.34 (0.95–1.89)	0.09
Death at 90 days (%)	46 (15.2)	71 (20.3)	0.05 (−0.01 to 0.11)	1.42 (0.94–2.13)	1.58 (1.03–2.43)	0.04

^a^ Values were adjusted for age, baseline NIHSS score, baseline ASPECTS, occlusion site, and time from last known well to randomization, as prespecified in the protocol and statistical analysis plan.

^b^ The β coefficient was estimated from a linear regression model.

^c^ Recanalization on follow-up CTA or MRA within 48 h data was not available for 78 and 94 patients in placebo group and tirofiban group, respectively.

^d^ Symptomatic ICH within 48 h data was not available for 1 patient in tirofiban group.

^e^ Any radiologic ICH within 48 h data was not available for 1 patient in tirofiban group.

CTA, computed tomography angiography; DSA, digital subtraction angiography; European Quality Five-Dimension Five-Level Self-Report Questionnaire (EQ-5D-5L); IQR, interquartile range; MRA, magnetic resonance angiography; NA, not applicable; NIHSS, National Institutes of Health Stroke Scale.

### Subgroup analysis

**Figure 4:**
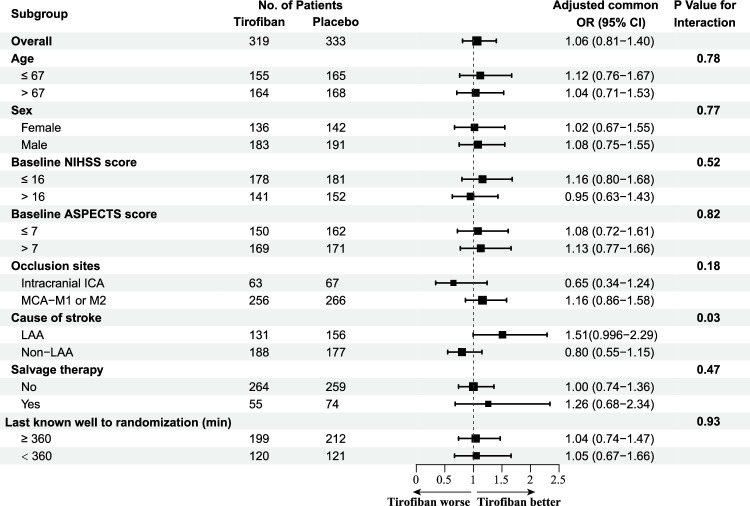
Heterogeneity of treatment effect among prespecified subgroups. Forest plot effect variation across eight prespecified subgroups for adjusted common odds ratio for less disability on the mRS at 90 days. ASPECTS, Alberta Stroke Program Early CT Score; ICA, internal carotid artery; LAA, large artery atherosclerosis; MCA-M1 or M2, first or second segment of middle cerebral artery; NIHSS, National Institutes of Health Stroke Scale. ^a^Salvage therapy was defined as failure of primary means of thrombectomy (e.g. stent-retriever or local aspiration) and balloon angioplasty and/or stenting was used.

**Figure 5:**
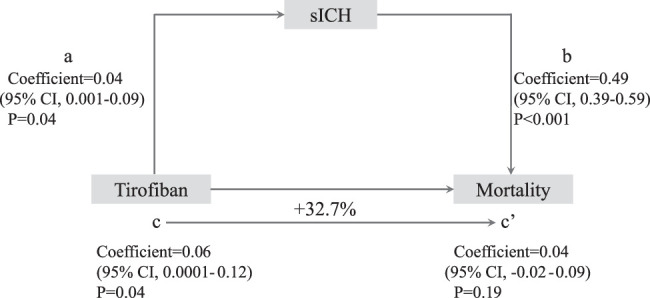
Diagram for the mediation analysis of the mediation effect of tirofiban on mortality via sICH in cardioembolism subgroup. a, regression coefficient of the association between tirofiban and sICH; b, regression coefficient of the association between sICH and mortality, using tirofiban, age, baseline NIHSS, baseline ASPECTS, time from onset to randomization, occlusion site and sICH as independent variables; c, regression coefficient of the association between tirofiban and mortality; c’, regression coefficient of the association between tirofiban and mortality, using tirofiban, age, baseline NIHSS, baseline ASPECTS, time from onset to randomization, occlusion site and sICH as independent variables. The percentage difference of the coefficients a*b/c is shown. sICH, symptomatic intracerebral hemorrhage; ASPECTS, Alberta Stroke Program Early CT Score; NIHSS, National Institutes of Health Stroke Scale.

The heterogeneity in the treatment effect of tirofiban was only observed for stroke etiology. Evidence of a potential benefit of tirofiban on the primary outcome was present for the LAA subgroup but not for the non-LAA subgroup, adjusted common ORs for less mRS disability 1.51 (95% CI: 0.996–2.29; *P*=0.052) versus 0.80 (95% CI: 0.55–1.15; *P*=0.23), *P* for interaction=0.03 (Fig. 3C and Fig. 4). Baseline characteristics, clinical efficacy, technical efficacy, and safety outcomes of the LAA subgroup (Supplementary Material Table S8, Supplemental Digital Content 3, http://links.lww.com/JS9/C664 and S9, Supplemental Digital Content 3, http://links.lww.com/JS9/C664) and cardioembolism (CE) subgroup (Supplementary Material Table S10, Supplemental Digital Content 3, http://links.lww.com/JS9/C664 and S11, Supplemental Digital Content 3, http://links.lww.com/JS9/C664) are shown in Supplement. Intravenous tirofiban might be associated with a lower level of disability [adjusted common ORs, 1.74 (95% CI: 1.14–2.65); *P*=0.01] among LAA patients, whereas intravenous tirofiban might be significantly increase sICH and mortality in CE patients. We performed a mediation analysis to investigate whether the effects of tirofiban on mortality were mediated by sICH. After including sICH as a mediator, we observed a significant partial mediation effect of sICH on the effects of tirofiban on the functional independence outcome in CE patients. The mediation effect of tirofiban was 0.06 (95% CI: 0.0001–0.12, *P*=0.04) with an estimated mediated proportion of 32.7% on mortality (Fig. 5).

## Discussion

These secondary analyses of the RESCUE BT trial yield three important new findings. First, intravenous tirofiban did not significantly improve the level of 90-day global disability in patients with AIS due to anterior circulation LVO in the overall trial populations who meeting the recent AHA/ASA guidelines. Second, intravenous tirofiban had a potential benefit in improving functional outcome but was not associated with decreased risk of early reocclusion rate of treated arteries in the LAA subgroup. Third, intravenous tirofiban increased the incidence of ICH, in particular significantly increased the incidence of sICH in patients with CE stroke, and appeared to lead to higher odds of death.

In this study, intravenous tirofiban administration did not confer significantly more benefit in the overall study populations. We found that tirofiban did not increase the proportion of final substantial reperfusion, which was consistent with previous findings^20^. Of note, the proportion of substantial reperfusion had reached to 90.7% in the placebo group, so the room for improvement of tirofiban to increase recanalization in this trial is very limited. Additionally, the incidence of early reocclusion was only 9.6% of patients in the placebo group, so tirofiban did not statistically significantly reduce the rate of reocclusion as expected. Previous studies have reported that early reocclusion occurred in 2.3–7.2% of patients with LVO undergoing EVT and was associated with early neurological deterioration, poor functional outcome, and increased mortality^21–23^. Current EVT strategies, which involve stent implantation, balloon angioplasty or endothelial injury in suspected cases, have effectively controlled the reocclusion rate. There was limited scope for tirofiban effect on reducing reocclusion of the LVO. In addition, intravenous tirofiban did not improve functional outcome after EVT, which may be related to the majority of non-LAA patients, especially 45.9% of patients with CE stroke. This was consistent with previous studies showing that patients with CE stroke had higher risk of sICH and worse clinical outcome after EVT due to there had poor collateral circulation and heavy thrombotic burden^24,25^.

The efficacy of tirofiban may be related to the etiology of AIS. In the current secondary analysis, although the proportion of successful recanalization was not increased and the proportion of reocclusion was not also reduced in patients with LAA stroke treated with tirofiban following EVT, the functional outcomes were much better. Futile recanalization or no reflow and microcirculation dysfunction could be a potential reason for this discrepancy. Even if the large artery occlusion could be successfully recanalized after EVT, microvascular thrombosis may still occur in the distal sites^26,27^. Given the topographical localization of microvascular distribution throughout the ischemic territory, in situ formation of microthrombosis and microembolism during EVT may consistently reduce blood flow and exacerbate infarct progression after ischemia^26,27^. Studies have shown that tirofiban could effectively prevent and treat microthrombosis and accelerate the recovery from AIS by altering the cerebrovascular microcirculation^27,28^. Therefore, we speculate that tirofiban may improve the functional outcome by improving the microvascular reperfusion status and reducing futile recanalization phenomenon in LAA patients.

Tirofiban has already been progressively applied in clinical practice as a potential agent to inhibit different stages of thrombosis mediated by activated platelets in AIS patients after EVT. Consistent with previous observational studies, tirofiban administration was associated with an increased risk of sICH and hemorrhagic transformation^25,29^. In LAA patients, the incidence of sICH did not differ between the tirofiban group and the placebo group, whereas tirofiban was associated with a 3.3-fold increase in sICH [10.8% versus 4.6%; adjusted ORs, 3.27 (95% CI: 1.24–8.61); *P*=0.02], which led to a 2.3-fold increase in mortality [20.9% versus 11.9%; adjusted ORs, 2.32 (95% CI: 1.20–4.51); *P*=0.01] in patients with cardioembolism. The heterogeneity of the actual thrombus composition may explain this discrepancy. It is known that vascular occlusions induced by cardiogenic emboli are rich in erythrocytes and are considered as red thrombi, whereas vascular occlusions induced by LAA are mainly composed of platelets and are referred to as white thrombi^24^. Therefore, tirofiban, as a nonpeptide glycoprotein IIb/IIIa receptor antagonist that can prevent platelet aggregation, is beneficial for LAA patients. Meanwhile, some patients with cardioembolic stroke in our study received antiplatelet or oral anticoagulant therapy prior to EVT, resulting in changes in blood status and hemodynamics. When tirofiban is reintroduced, the dynamic balance between coagulation and anticoagulation is disrupted and the risk of sICH is increased. Although studies have shown that aspirin and warfarin (if INR≤1.7) do not increase the risk of hemorrhage^24,30^, the subsequent use of tirofiban after EVT may increase the risk of hemorrhage. Therefore, tirofiban could be recommended for AIS patients with LAA, whereas tirofiban may not be suitable for CE patients due to lack of efficacy. In addition, the EVT operation is relatively complicated in cardioembolic stroke patients due to the heavy thrombus burden, and the risk of sICH is inevitably increased. Of course, the infarct core volume in patients with cardioembolic stroke may also be a potential risk factor for sICH. However, whether tirofiban treatment may adversely affect outcome of CE patients treated with EVT would be of interest for future research.

Our data are consistent with the main paper including all participants and suggest that intravenous tirofiban prior to endovascular thrombectomy in patients with ischemic stroke who meeting the criteria for DAWN and DEFUSE-3 did not increase the clinical benefits and may instead increase the incidence of any ICH, even sICH^9^. However, the safety and efficacy of tirofiban were not demonstrated in the ineligible participants of DAWN and DEFUSE-3 criteria. Therefore, the efficacy and safety of intravenous tirofiban before endovascular thrombectomy may depend on the optimizing patient selection according to the DAWN and DEFUSE-3 criteria in an extended time window.

A strength of this study lies in its large-scale, placebo-controlled, and double-blind design. The safety and potential efficacy of intravenous tirofiban administration during EVT in AIS patients has been preliminarily confirmed. Additionally, a major strength is lies in optimizing patient selection using advanced imaging, recruiting patients within a relatively broad treatment window of 24 h according to the DAWN and DEFUSE-3 enrollment criteria, as reported in the current AHA/ASA and European Stroke Organization guidelines.

### Limitations

This study has several limitations. First, this study was designed to enroll patients with LVO for simple randomization by treatment (tirofiban or placebo), but not for stratified randomization based on etiology (LAA or CE). To generalize the clinical application of this study, randomized trials of intravenous tirofiban or placebo by stroke etiology are needed. Secondly, tirofiban was administered at a fixed-dosage rather than in a dose-escalation study, which may confound its therapeutic effects at specific doses. Therefore, further dose-escalation studies are needed to determine the optimal dosage regimen. Thirdly, this is a secondary analysis with selection bias as the study population was selected from the RESCUE BT trial according to the current AHA/ASA guidelines. The treatment contrast in different time windows (early time window and extended time window) was not randomized. Although we adjusted for multiple potential confounders, selection bias still remains and is likely to affect the results of the analysis. Fourth, we did not analyze the difference in DOAC concentration between the two groups in patients with a history of anticoagulant treatment and acute LVO. Laboratory monitoring of coagulation parameters alone cannot accurately assess the anticoagulant activity and ICH risk associated with DOACs and may introduce bias in the occurrence of bleeding complications. Fifth, the patients in the RESCUE BT trial relied on noninvasive imaging prior to EVT to determine the exact cause of LVO, which can be challenging and were not stratified and randomized according to stroke subtypes, such as CE versus other etiologies. Therefore, there may have been heterogeneity in stroke subtypes within the CE subgroup, which could have influenced the treatment response to tirofiban. Finally, because the primary analysis of this study was under powered, it should be very cautious to conclude findings from the sensitivity or subgroup analyses. these results should be interpreted with caution.

## Conclusion

Optimizing patient selection with advanced imaging profiles according to current guidelines remained did not alter the effect of intravenous tirofiban combined with EVT on functional outcomes in AIS patients with LVO in anterior circulation. The findings do not support periprocedural intravenous tirofiban for AIS. Although periprocedural intravenous tirofiban was strongly associated with functional improvement, which shown the potential for periprocedural adjuvant therapy in patients with LAA following EVT, but there was an increased risk of sICH in patients with cardioembolism stroke, which needs to be more cautious in the clinical practice. Further randomized controlled trials are warranted to validate the present results.

## Ethical approval

The study was approved by the ethics committees of the Chinese Ethics Committee of Registering Clinical Trials (ChiECRCT-20180012), and all participating centers prior to the start of the study, and all enrolled patients or their legally authorized representatives provided written informed consent before enrollment.

## Consent

The study was approved by the all participating centers prior to the start of the study, and all enrolled patients or their legally authorized representatives provided written informed consent before enrollment.

## Sources of funding

The trial was supported by Lunan Pharmaceutical Group Co., Ltd., China, National Natural Science Foundation of China (No. 82201469, 82271349) and Chongqing Science and Health Joint Project (2024MSXM006), The Youth Doctoral Talent Incubation Program of the Second Affiliated Hospital of the Army Medical University (2022YQB011).

## Author contribution

W.L, L.F., and K.W.: conceptualized and designed the study; K.W., L.F., and Z.W.: had full access to all the data in the study and take responsibility for the integrity of the data and the accuracy of the data analysis; Drs W.L., H.J., S.J.: contributed equally; K.W. and H.J.: performed the data analysis; W.L, K.W., H.J., S.J., L.F., and Z.W.: drafted the manuscript; Y.Q.: contributed to revision of the manuscript and approved the final draft. All authors contributed to the data acquisition, interpretation, intellectual content, critical revision to the drafts of the paper and approved the final version.

## Conflicts of interest disclosure

The authors declare that they have no conflicts of interest.

## Research registration unique identifying number (UIN)

URL: http://www.chictr.org.cn/; Registration number: ChiCTR-INR-17014167, Registered 27 December 2017.

## Guarantor

Weilin Kong and Fengli Li had primary responsibility for the final content.

## Data availability statement

Data are available from the corresponding author upon reasonable requests.

## Provenance and peer review

This paper was not invited.

## Supplementary Material
