# Supplementary figures and images for: Association of functional outcomes between intravenous tirofiban and endovascular thrombectomy in imaging-screened patients with large vessel occlusion stroke: a secondary analysis of randomized clinical trial
Source: Int J Surg. 2024 May 24;110(9):5505–17. doi: 10.1097/JS9.0000000000001666 (PMC11392134; doi:10.1097/JS9.0000000000001666)

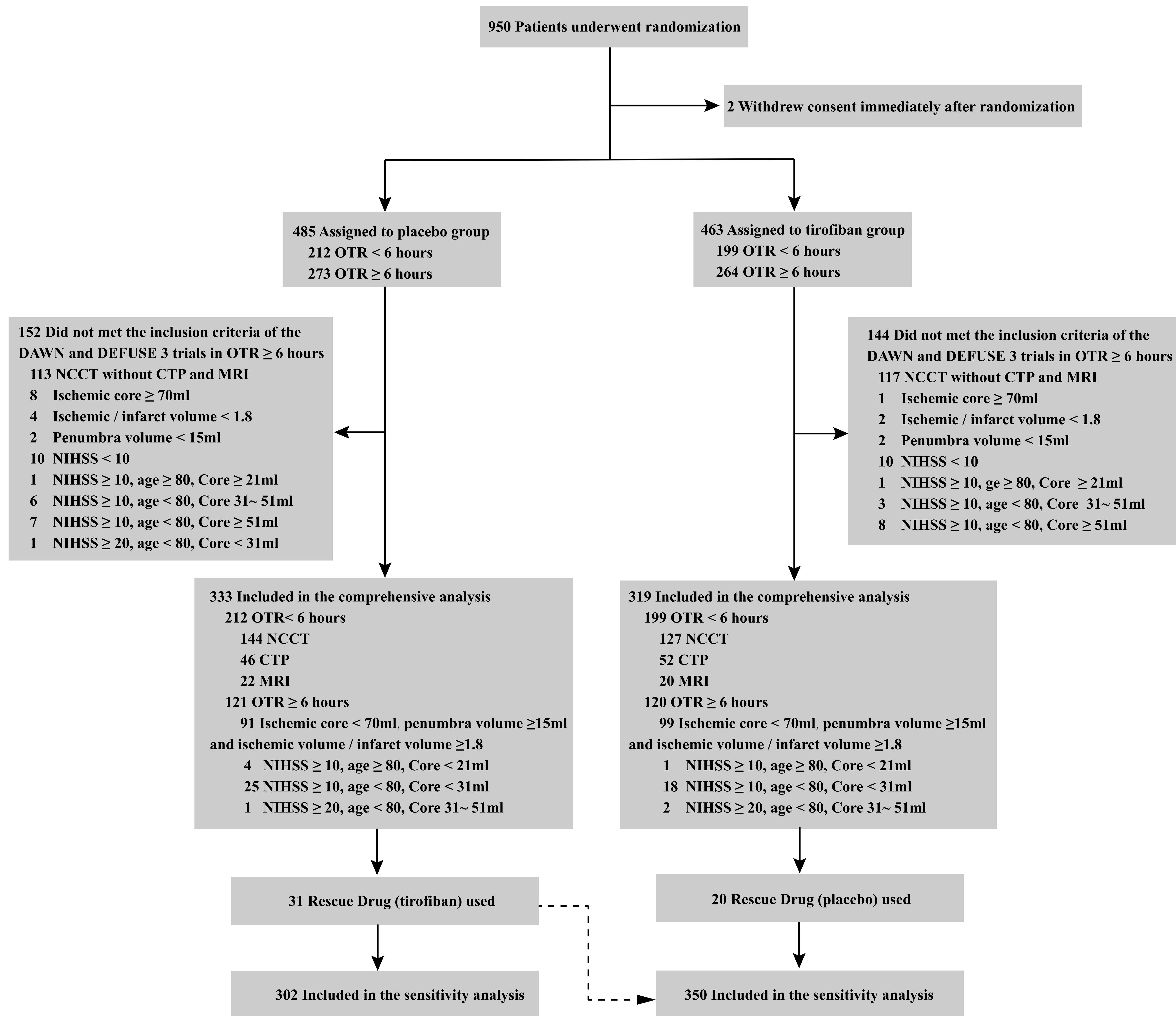

Supplement: Supplementary file 2 [file js9-110-5505-s002.pdf]
