# Supplementary material for: Association of functional outcomes between intravenous tirofiban and endovascular thrombectomy in imaging-screened patients with large vessel occlusion stroke: a secondary analysis of randomized clinical trial
Source: Int J Surg. 2024 May 24;110(9):5505–17. doi: 10.1097/JS9.0000000000001666 (PMC11392134; doi:10.1097/JS9.0000000000001666)
Supplement: Supplementary file 3 [file js9-110-5505-s003.docx]

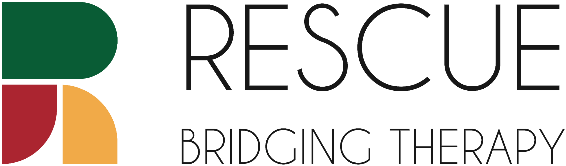


**Association of functional outcomes between intravenous tirofiban and endovascular thrombectomy in imaging-screened patients with large vessel occlusion stroke: a secondary analysis of randomized clinical trial**

**SUPPLEMENTARY MATERIAL**

This material has been provided by the authors to give readers additional information about their work.

**Contents**

Supplementary Methods 3

Supplementary Figures 4

Figure S1. Heterogeneity of treatment effect in AHA/ASA Guideline-Eligible participants or AHA Guideline-Ineligible participants with large vessel occlusion stroke. 4

Figure S2. Kaplan-Meier estimates of the probability of death in overall patients. 5

Figure S3. Tirofiban Treatment Effect and Probability of mortality. 6

Figure S4. Tirofiban Treatment Effect and Probability of sICH. 7

Supplementary Tables 8

Table S1. Baseline Characteristics according to treatment group in AHA/ASA Guideline-Ineligible participants with large vessel occlusion stroke 8

Table S2. Baseline Characteristics according to AHA/ASA Guideline-Eligible Patients and AHA/ASA Guideline-Ineligible Patients in participants with large vessel occlusion stroke 11

Table S3. Primary and Secondary Efficacy Outcomes and Safety Outcomes according to treatment group in in AHA/ASA Guideline-Ineligible participants with large vessel occlusion stroke 13

Table S4. Adverse events and complication according to treatment group in participants with large vessel occlusion stroke 15

Table S5. Baseline Characteristics and workflow measures of the Patients and Features in sensitivity analysis 16

Table S6. Efficacy Outcomes and Primary Safety Outcomes in sensitivity analysis 19

Table S7. Adverse events and complications in sensitivity analysis 21

Table S8. Baseline Characteristics and workflow measures of the Patients in large artery atherosclerosis stratum 22

Table S9. Analysis of clinical and safety outcomes in large artery atherosclerosis stratum 25

Table S10. Baseline Characteristics and workflow measures of the Patients in cardioembolic stroke stratum 28

Table S11. Analysis of clinical and safety outcomes in cardioembolic stroke stratum 31

# Supplementary Methods

Image Analysis

An imaging core laboratory independently evaluated all available imaging data. The Fast-Processing of Ischemic Stroke software (version 1.0.22), an automated image postprocessing system approved by the Chinese National Medical Products Administration was used to evaluate the baseline ASPECTS on NCCT as well as the CTP or MRI perfusion imaging data from participating centers. The ischemic core was defined as a relative cerebral blood flow <30% of the contralateral normal tissue or an apparent diffusion coefficient <620 μm2/s on MRI. Critically hypo-perfused tissue (ie, penumbra) was identified by the perfusion parameter: time to maximum contrast intensity of the tissue residue function >6 s lesion volume. The mismatch volume was calculated as the critically hypo-perfused tissue volume minus the ischemic core tissue volume. The CTP mismatch ratio was calculated by ratios between the ischemic stroke core and penumbra volumes. The Fast-Processing of Ischemic Stroke has demonstrated excellent agreement with the RAPID software in assessing the volumes of both the relative cerebral blood flow <30% and the time to maximum contrast intensity of the tissue residue function >6 s perfusion lesions. The Fast-Processing of Ischemic Stroke software was provided to centers under a limited research license.

# Supplementary Figures

## Figure S1. Heterogeneity of treatment effect in AHA/ASA Guideline-Eligible participants or AHA Guideline-Ineligible participants with large vessel occlusion stroke.


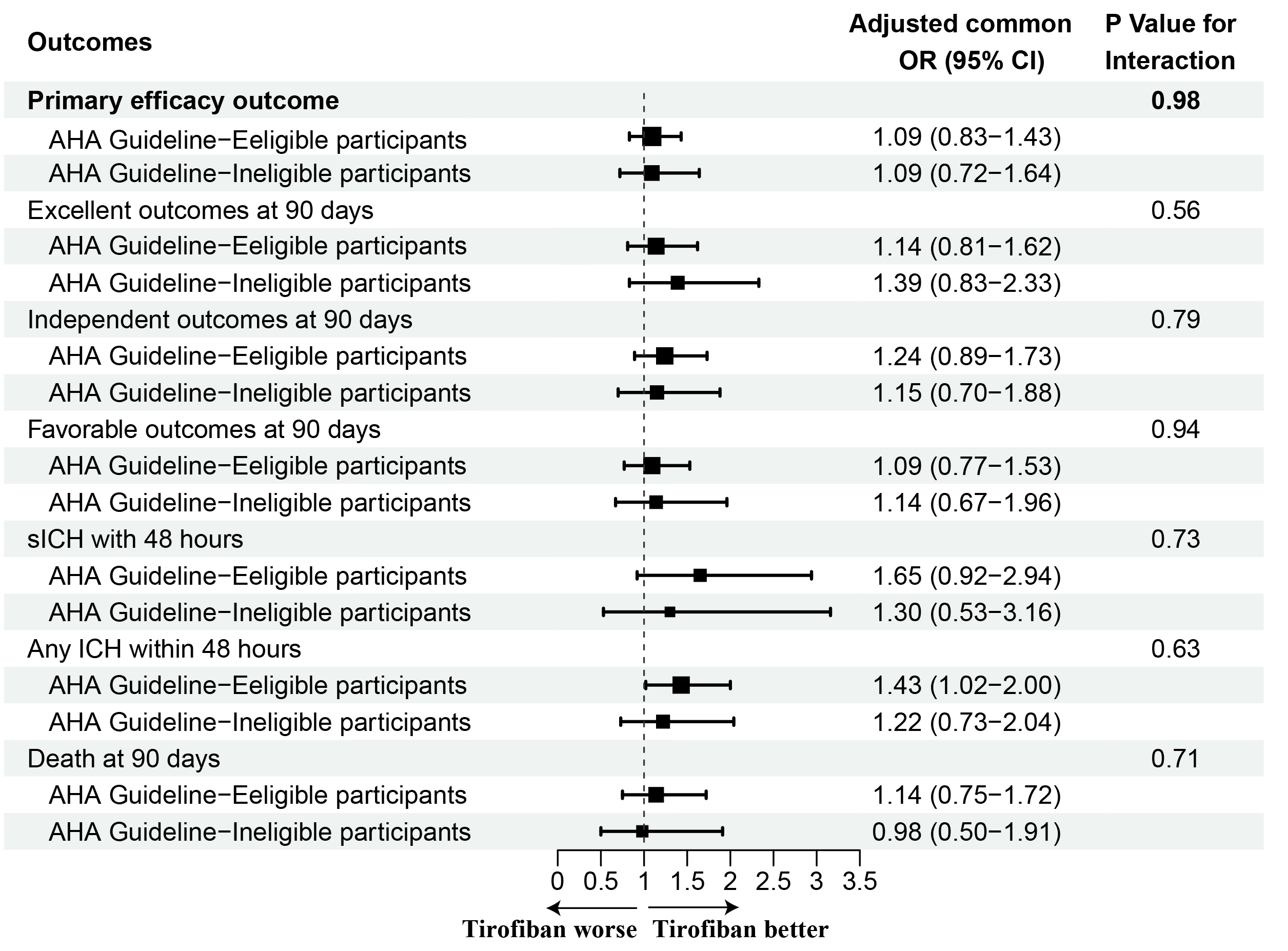


Ordinal and logistic regression models with including treatment effect adjusted for age, baseline NIHSS, baseline ASPECTS, time from onset to randomization, occlusion site. OR indicates odds ratio.

## Figure S2. Kaplan-Meier estimates of the probability of death in overall patients.


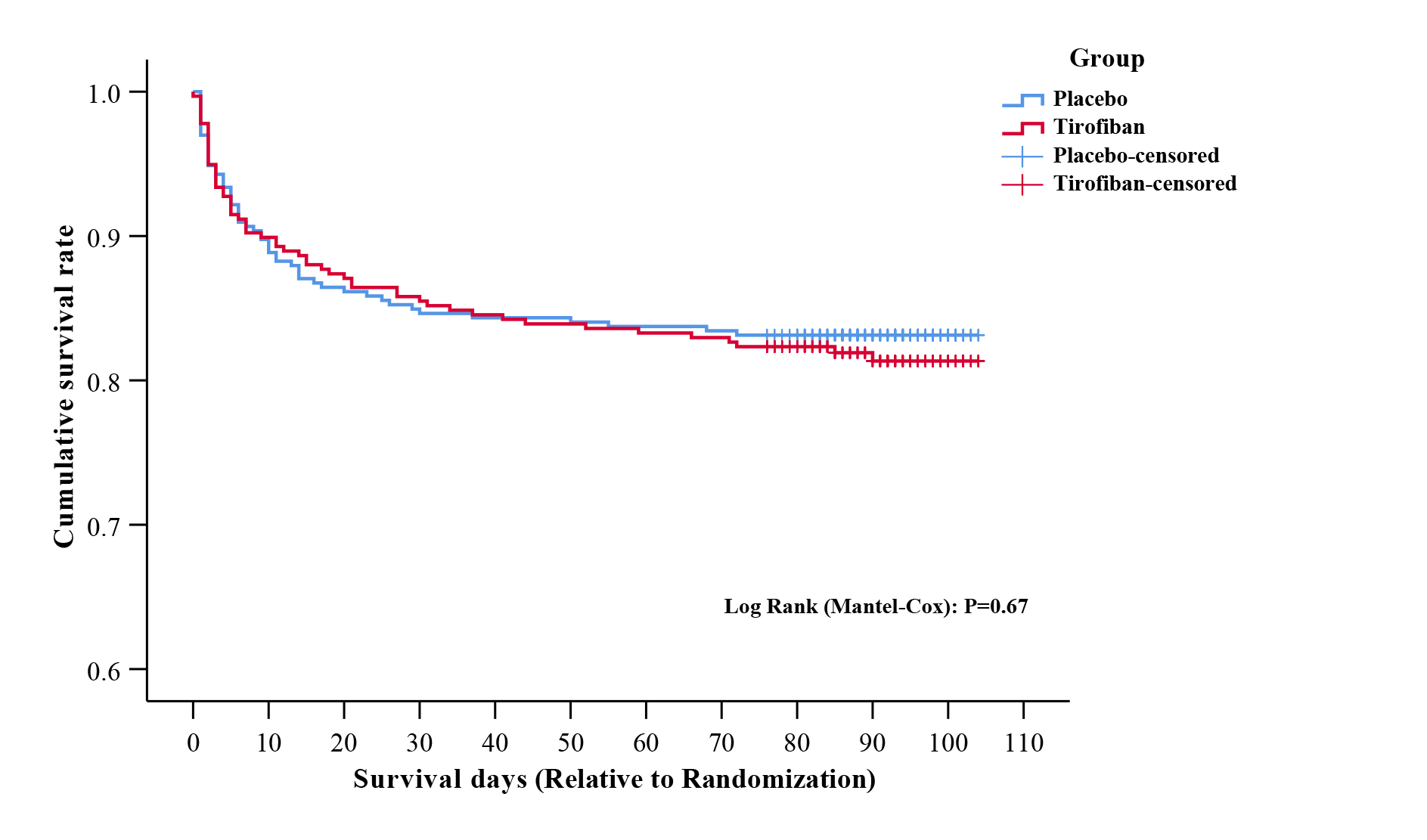


Log Rank (Mantel-Cox): P = 0.67

## Figure S3. Tirofiban Treatment Effect and Probability of mortality.


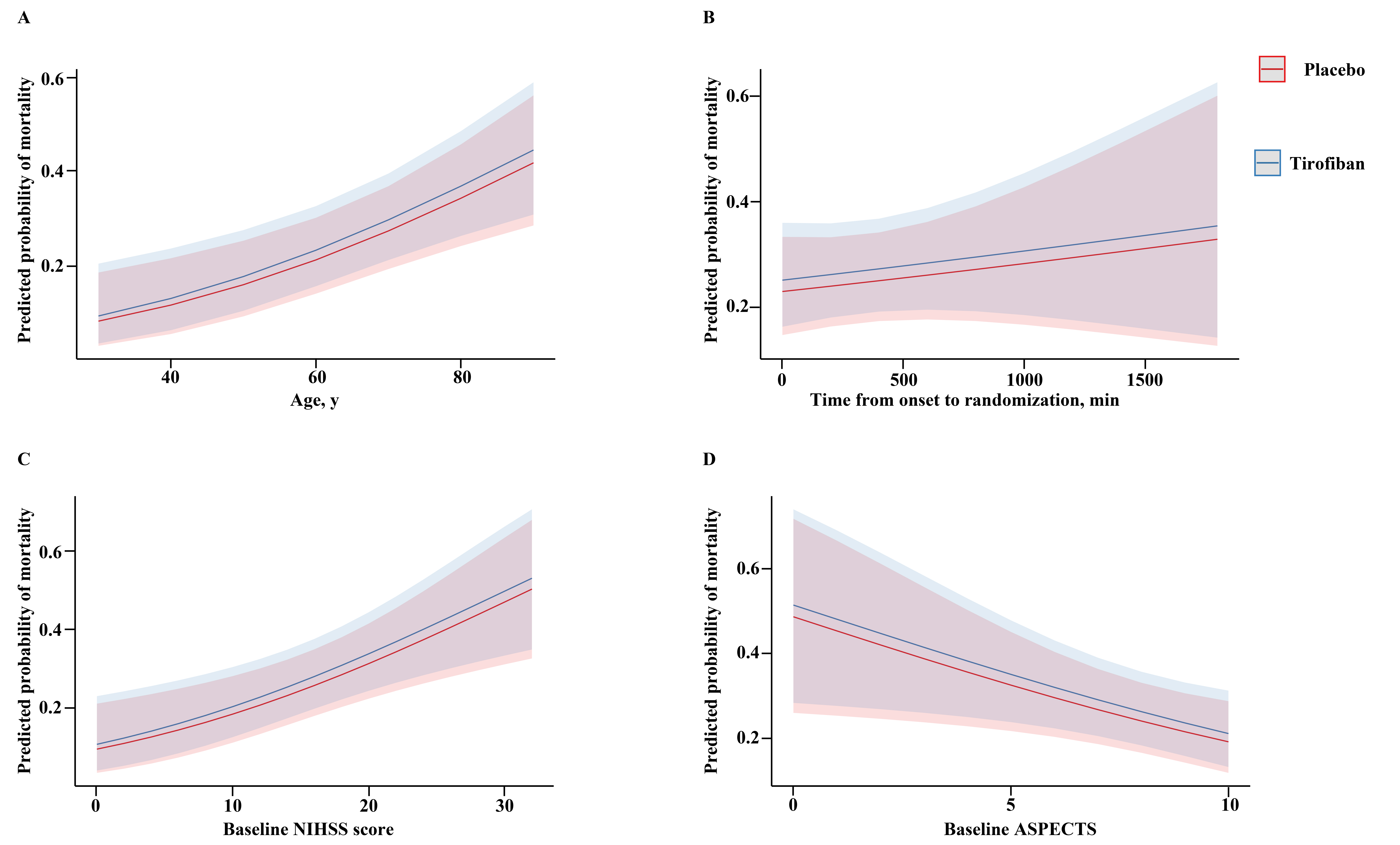


The 4 graphs show the probability and 95% CI of mortality at 90 days, separately for patients in the placebo arm (light red lines and shading) and the tirofiban treatment arm (light blue lines and shading). The probability of mortality was not associated with age (A), time from onset to randomization (B), baseline NIHSS score (C) and baseline ASPECTS (D).

ASPECTS indicates Alberta Stroke Program Early CT Score; NIHSS indicates National Institutes of Health Stroke Scale.

## Figure S4. Tirofiban Treatment Effect and Probability of sICH.


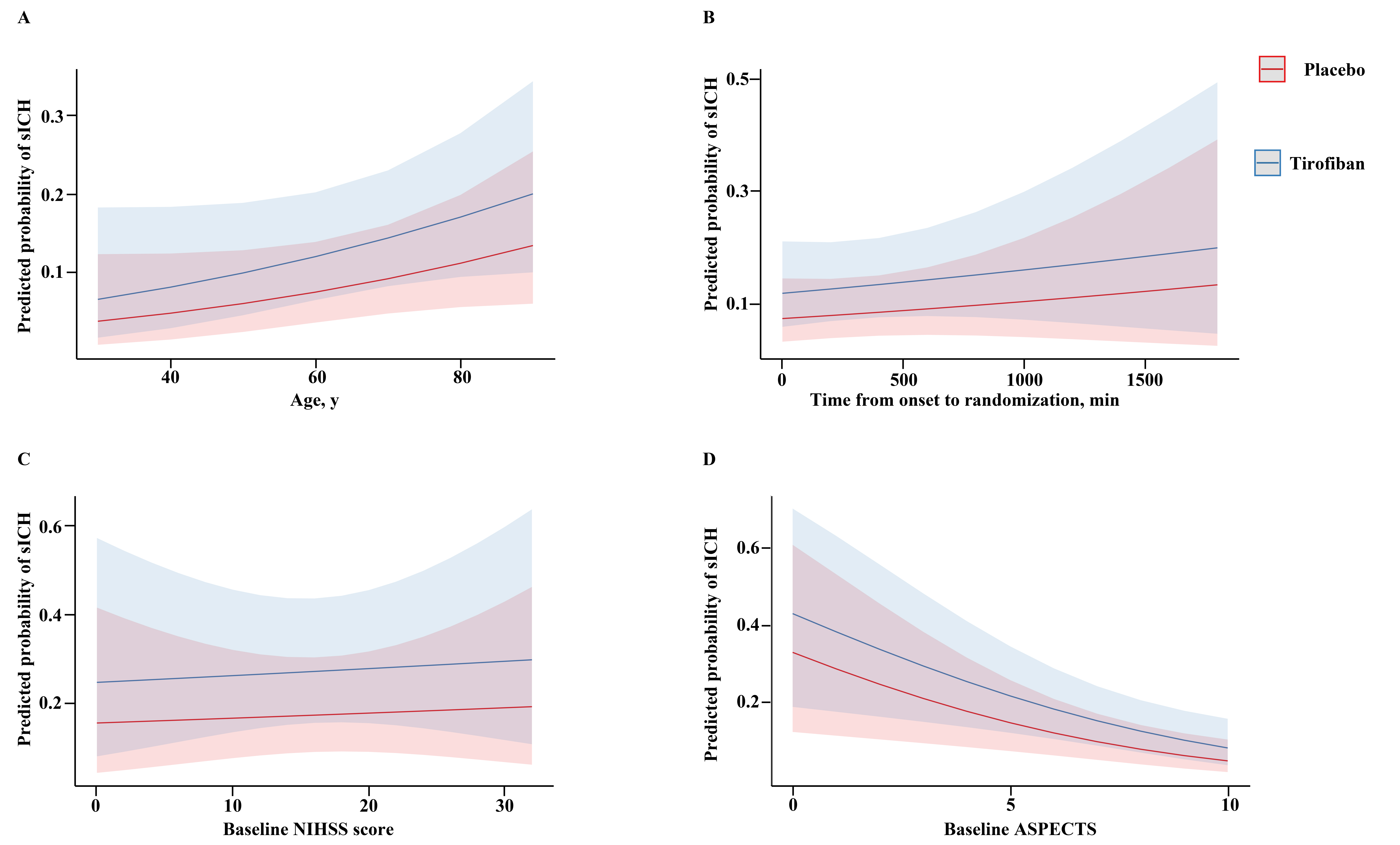


The 4 graphs show the probability and 95% CI of sICH within 48 hours, separately for patients in the placebo arm (light red lines and shading) and the tirofiban treatment arm (light blue lines and shading). The probability of mortality was not associated with age (A), time from onset to randomization (B), baseline NIHSS score (C) and baseline ASPECTS (D).

sICH indicates symptomatic intracerebral hemorrhage; ASPECTS indicates Alberta Stroke Program Early CT Score; NIHSS indicates National Institutes of Health Stroke Scale.

# Supplementary Tables

## Table S1. Baseline Characteristics according to treatment group in AHA/ASA Guideline-Ineligible participants with large vessel occlusion stroke

| Characteristic | Overall  (n=296) | Placebo group (n=152) | Tirofiban group (n=144) | P value |
| --- | --- | --- | --- | --- |
| Demographic characteristics |  |  |  |  |
| Age, median (IQR) | 66 (56-74) | 65 (55-73) | 68 (56-74) | 0.16 |
| Sex, no. (%) |  |  |  | 0.03 |
| Women | 113 (38.2) | 49 (32.2) | 64 (44.4) |  |
| Men | 183 (61.8) | 103 (67.8) | 80 (55.6) |  |
| Medical history, no. (%) ^a^ |  |  |  |  |
| Hypertension | 153 (51.7) | 83 (54.6) | 70 (48.6) | 0.30 |
| Diabetes | 52 (17.6) | 31 (20.4) | 21 (14.6) | 0.19 |
| Hyperlipidemia | 40 (13.5) | 14 (9.2) | 26 (18.1) | 0.03 |
| Atrial fibrillation | 81 (27.4) | 33 (21.7) | 48 (33.3) | 0.03 |
| Ischemic stroke | 49 (16.6) | 32 (21.1) | 17 (11.8) | 0.03 |
| Smoking (Current or Past) | 75 (25.3) | 43 (28.3) | 32 (22.2) | 0.23 |
| Prestroke modified Rankin scale score |  |  |  | 0.13 |
| 0 | 274 (92.6) | 136 (89.5) | 138 (95.8) |  |
| 1 | 16 (5.4) | 12 (7.9) | 4 (2.8) |  |
| 2 | 5 (1.7) | 3 (2.0) | 2 (1.4) |  |
| 3 | 1 (0.3) | 1 (0.7) | 0 (0.0) |  |
| 4 | 0 (0.0) | 0 (0.0) | 0 (0.0) |  |
| Stroke etiology, no. (%) |  |  |  | 0.02 |
| Large artery atherosclerosis | 148 (50.0) | 82 (53.9) | 66 (45.8) |  |
| Cardioembolism | 107 (36.1) | 43 (28.3) | 64 (44.4) |  |
| Unknown | 12 (4.1) | 7 (4.6) | 5 (3.5) |  |
| Other | 29 (9.8) | 20 (13.2) | 9 (6.3) |  |
| Clinical characteristics |  |  |  |  |
| Baseline NIHSS score, median (IQR) | 15 (11-19) | 14 (11-20) | 15 (11-19) | 0.67 |
| Baseline Systolic blood pressure, median (IQR), mmHg | 145 (129-164) | 146 (129-160) | 145 (127-168) | 0.74 |
| Baseline Diastolic blood pressure, median (IQR), mmHg | 84 (74-95) | 86 (74-98) | 84 (75-92) | 0.35 |
| Baseline Serum glucose, median (IQR), mmol/L ^b^ | 6.9 (5.7-8.6) | 6.7 (5.6-8.8) | 7.0 (5.7-8.5) | 0.71 |
| Imaging characteristics, no. (%) |  |  |  | 0.12 |
| NCCT | 230 (77.7) | 113 (74.3) | 117 (81.3) |  |
| CTP | 19 (6.4) | 14 (9.2) | 5 (3.5) |  |
| MRI | 47 (15.9) | 25 (16.4) | 22 (15.3) |  |
| Baseline ASPECTS, median (IQR) | 8 (7-9) | 7 (7-9) | 8 (7-9) | 0.33 |
| Occlusion site, no. (%) |  |  |  | 0.85 |
| ICA intracranial | 64 (21.6) | 31 (20.4) | 33 (22.9) |  |
| Middle cerebral artery |  |  |  |  |
| M1 segment | 191 (64.5) | 99 (65.1) | 92 (63.9) |  |
| M2 segment | 41 (13.9) | 22 (14.5) | 19 (13.2) |  |
| Collateral status, no. (%) |  |  |  | 0.73 |
| ASITN/SIR grade 0 | 17 (5.7) | 9 (5.9) | 8 (5.6) |  |
| ASITN/SIR grade 1 | 58 (19.6) | 29 (19.1) | 29 (19.6) |  |
| ASITN/SIR grade 2 | 134 (45.3) | 65 (42.8) | 69 (47.9) |  |
| ASITN/SIR grade 3 | 84 (28.4) | 48 (31.6) | 36 (25.0) |  |
| ASITN/SIR grade 4 | 3 (1.0) | 1 (0.7) | 2 (1.4) |  |
| Tandem lesion, no. (%) |  |  |  | 0.89 |
| No | 280 (94.6) | 143 (94.1) | 137 (95.1) |  |
| Severe stenosis of extracranial segment (>=70%) | 14 (4.7) | 8 (5.3) | 6 (4.2) |  |
| Extracranial occlusion | 2 (0.7) | 1 (0.7) | 1 (0.7) |  |
| Total passes, median (IQR) | 1 (1-2) | 2 (1-2) | 1 (1-2) | 0.14 |
| First pass effect, no. (%) | 77 (26.0) | 32 (21.1) | 45 (31.3) | 0.046 |
| Stent thrombectomy only, no. (%) | 38 (12.8) | 16 (10.5) | 22 (15.3) | 0.22 |
| Aspiration only, no. (%) | 58 (19.6) | 24 (15.8) | 34 (23.6) | 0.09 |
| Salvage therapy, no. (%) | 70 (23.6) | 41 (27.0) | 29 (20.1) | 0.17 |
| SWIM only, no. (%) | 40 (13.5) | 21 (13.8) | 19 (13.2) | 0.88 |
| Anesthesia, no. (%) |  |  |  | 0.73 |
| General | 85 (28.7) | 45 (29.6) | 40 (27.8) |  |
| Local | 211 (71.3) | 107 (70.4) | 104 (72.2) |  |
| Onset to randomization, median (IQR), min | 555 (439-750) | 561 (438-728) | 554 (446-769) | 0.69 |
| Door to puncture, median (IQR), min | 115 (85-163) | 117 (89-157) | 113 (78-169) | 0.66 |
| Puncture to recanalization, median (IQR), min | 65 (43-105) | 70 (45-118) | 60 (40-89) | 0.11 |

Abbreviations: IQR, interquartile range; EVT, endovascular therapy; NCCT, non-contrast computed tomography; CTP, computed tomography perfusion; MRI, magnetic resonance imaging; ASPECTS, Alberta Stroke Program Early CT Score; ASITN/SIR, American Society of Intervention and Therapeutic Neuroradiology/Society of Interventional Radiology; NIHSS, National Institutes of Health Stroke Scale; ICA, internal carotid artery.

^a^ Patient self-report or family report.

^b^ Serum glucose data was not available for 13 and 12 patients in placebo group and tirofiban group, respectively.

## Table S2. Baseline Characteristics according to AHA/ASA Guideline-Eligible Patients and AHA/ASA Guideline-Ineligible Patients in participants with large vessel occlusion stroke

| Characteristic | AHA Guideline-Eligible Patients (n=652) | AHA Guideline-Ineligible Patients  (n=296) | P value |
| --- | --- | --- | --- |
| Demographic characteristics |  |  |  |
| Age, median (IQR) | 68 (58-75) | 66 (56-74) | 0.04 |
| Sex, no. (%) |  |  | 0.20 |
| Women | 278 (42.6) | 113 (38.2) |  |
| Men | 374 (57.4) | 183 (61.8) |  |
| Medical history, no. (%) ^a^ |  |  |  |
| Hypertension | 371 (56.9) | 153 (51.7) | 0.14 |
| Diabetes | 152 (23.3) | 52 (17.6) | 0.046 |
| Hyperlipidemia | 92 (14.1) | 40 (13.5) | 0.81 |
| Atrial fibrillation | 232 (35.6) | 81 (27.4) | 0.01 |
| Ischemic stroke | 112 (17.2) | 49 (16.6) | 0.71 |
| Smoking (Current or Past) | 147 (22.5) | 75 (25.3) | 0.41 |
| Prestroke modified Rankin scale score |  |  | 0.77 |
| 0 | 592 (90.8) | 274 (92.6) |  |
| 1 | 42 (6.4) | 16 (5.4) |  |
| 2 | 16 (2.5) | 5 (1.7) |  |
| 3 | 1 (0.2) | 1 (0.3) |  |
| 4 | 1 (0.2) | 0 (0.0) |  |
| Stroke etiology, no. (%) |  |  | 0.04 |
| Large artery atherosclerosis | 287 (44.0) | 148 (50.0) |  |
| Cardioembolism | 299 (45.9) | 107 (36.1) |  |
| Unknown | 48 (7.4) | 12 (4.1) |  |
| Other | 18 (2.8) | 29 (9.8) |  |
| Clinical characteristics |  |  |  |
| Baseline NIHSS score, median (IQR) | 16 (12-19) | 15 (11-19) | 0.15 |
| Baseline Systolic blood pressure, median (IQR), mmHg | 145 (130-160) | 145 (129-164) | 0.46 |
| Baseline Diastolic blood pressure, median (IQR), mmHg | 84 (76-94) | 84 (74-95) | 0.97 |
| Baseline Serum glucose, median (IQR), mmol/L ^b^ | 6.9 (5.8-8.7) | 6.9 (5.7-8.6) | 0.55 |
| Imaging characteristics, no. (%) |  |  | <0.001 |
| NCCT | 271 (41.6) | 230 (77.7) |  |
| CTP | 288 (44.2) | 19 (6.4) |  |
| MRI | 93 (14.3) | 47 (15.9) |  |
| Baseline ASPECTS, median (IQR) | 8 (7-9) | 8 (7-9) | 0.74 |
| Occlusion site, no. (%) |  |  | 0.83 |
| ICA intracranial | 130 (19.9) | 64 (21.6) |  |
| Middle cerebral artery |  |  |  |
| M1 segment | 424 (65.0) | 191 (64.5) |  |
| M2 segment | 98 (15.0) | 41 (13.9) |  |
| Collateral status, no. (%) ^c^ |  |  | 0.35 |
| ASITN/SIR grade 0 | 54 (8.3) | 17 (5.7) |  |
| ASITN/SIR grade 1 | 151 (23.2) | 58 (19.6) |  |
| ASITN/SIR grade 2 | 261 (40.1) | 134 (45.3) |  |
| ASITN/SIR grade 3 | 179 (27.5) | 84 (28.4) |  |
| ASITN/SIR grade 4 | 6 (0.9) | 3 (1.0) |  |
| Tandem lesion, no. (%) |  |  | 0.26 |
| No | 617 (94.6) | 280 (94.6) |  |
| Severe stenosis of extracranial segment (>=70%) | 25 (3.8) | 14 (4.7) |  |
| Extracranial occlusion | 10 (1.5) | 2 (0.7) |  |
| Total passes, median (IQR) | 2 (1-2) | 1 (1-2) | 0.15 |
| First pass effect, no. (%) | 178 (27.3) | 77 (26.0) | 0.76 |
| Stent thrombectomy only, no. (%) | 93 (14.3) | 38 (12.8) | 0.65 |
| Aspiration only, no. (%) | 126 (19.3) | 58 (19.6) | 0.92 |
| Salvage therapy, no. (%) | 129 (19.8) | 70 (23.6) | 0.22 |
| SWIM only, no. (%) | 119 (18.3) | 40 (13.5) | 0.07 |
| Anesthesia, no. (%) |  |  | 0.06 |
| General | 226 (34.7) | 85 (28.7) |  |
| Local | 426 (65.3) | 211 (71.3) |  |
| Onset to randomization, median (IQR), min | 386 (283-619) | 555 (439-750) | <0.001 |
| Door to puncture, median (IQR), min | 105 (78-144) | 115 (85-163) | 0.006 |
| Puncture to recanalization, median (IQR), min | 70 (40-108) | 65 (43-105) | 0.56 |

Abbreviations: IQR, interquartile range; EVT, endovascular therapy; NCCT, non-contrast computed tomography; CTP, computed tomography perfusion; MRI, magnetic resonance imaging; ASPECTS, Alberta Stroke Program Early CT Score; ASITN/SIR, American Society of Intervention and Therapeutic Neuroradiology/Society of Interventional Radiology; NIHSS, National Institutes of Health Stroke Scale; ICA, internal carotid artery.

^a^ Patient self-report or family report.

^b^ Serum glucose data was not available for 41 and 25 patients in AHA Guideline-Eligible Patients group and AHA Guideline-Ineligible Patients group, respectively.

^c^ ASITN/SIR by DSA data was not available for 1 patient AHA Guideline-Eligible Patients group.

## Table S3. Primary and Secondary Efficacy Outcomes and Safety Outcomes according to treatment group in in AHA/ASA Guideline-Ineligible participants with large vessel occlusion stroke

| Characteristic | Placebo group (n=152) | Tirofiban group (n=144) | Unadjusted difference (95% CI) | Unadjusted OR (95% CI) | Adjusted OR (95% CI) ^a^ | P value |
| --- | --- | --- | --- | --- | --- | --- |
| Primary efficacy outcome |  |  |  |  |  |  |
| mRS score at 90 days, median (IQR) | 3 (1-4) | 3 (1-4) | 0 (0 to 0) | 1.07 (0.72-1.59) | 1.09 (0.72-1.64) | 0.68 |
| Secondary efficacy outcomes |  |  |  |  |  |  |
| Excellent outcomes at 90 days (%) | 51 (33.6) | 56 (38.9) | 0.05 (-0.06 to 0.16) | 1.26 (0.78-2.03) | 1.38 (0.82-2.32) | 0.23 |
| Independent outcomes at 90 days (%) | 70 (46.1) | 69 (47.9) | 0.02 (-0.09 to 0.13) | 1.08 (0.68-1.70) | 1.14 (0.69-1.88) | 0.60 |
| Favorable outcomes at 90 days (%) | 94 (61.8) | 90 (62.5) | 0.01 (-0.10 to 0.12) | 1.03 (0.64-1.65) | 1.15 (0.67-1.97) | 0.61 |
| NIHSS change from baseline to 24 hours, median (IQR) ^b^ | -2 (-6 to 2) | -1 (-5 to 1) | 0 (-1 to 2) | 0.28 (-1.58 to 2.14) | 0.17 (-1.66 to 2.00) | 0.67 |
| NIHSS change from baseline to 5~7 days or early discharge, median (IQR) ^b^ | -4 (-9 to 1) | -4 (-9 to 2) | 0 (-2 to 2) | 0.68 (-1.71 to 3.07) | 0.45 (-1.87-2.76) | 0.70 |
| EQ-5D-5L score at 90d, median (IQR) ^b^ | 0.7 (0.1-1.0) | 0.7 (0.2-1.0) | 0 (0 to 0.05) | 0.03 (-0.06 to 0.12) | 0.04 (-0.04 to 0.12) | 0.35 |
| Secondary technical efficacy outcomes |  |  |  |  |  |  |
| Substantial reperfusion on initial DSA prior to EVT (%) | 2 (1.3) | 0 (0.0) | -0.01 (-0.05 to 0.01) | NA | NA | NA |
| Substantial reperfusion at final angiogram EVT (%) | 137 (90.1) | 135 (93.8) | 0.04 (-0.03 to 0.10) | 1.64 (0.70-3.88) | 1.60 (0.67-3.86) | 0.29 |
| Recanalization on follow-up CTA or MRA within 48 hours (%) ^c^ | 105 (87.5) | 97 (93.3) | 0.06 (-0.02 to 0.14) | 1.98 (0.77-5.06) | 2.27 (0.85-6.05) | 0.10 |
| Primary safety outcomes |  |  |  |  |  |  |
| Symptomatic ICH within 48 hours (%) ^d^ | 10 (6.7) | 13 (9.0) | 0.02 (-0.04 to 0.09) | 1.39 (0.59-3.28) | 1.24 (0.50-3.03) | 0.64 |
| Any radiologic ICH within 48 hours (%) ^e^ | 42 (28.0) | 47 (32.6) | 0.05 (-0.05 to 0.15) | 124 (0.76-2.05) | 1.21 (0.72-2.03) | 0.47 |
| Death at 90 days (%) | 25 (16.4) | 24 (16.7) | 0.002 (-0.08 to 0.09) | 1.02 (0.55-1.88) | 0.99 (0.51-1.94) | 0.99 |

CIs, confidence intervals; EVT, endovascular therapy; CTA, computed tomography angiography; DSA, digital subtraction angiography; IQR, interquartile range; MRA, magnetic resonance angiography; NA, not applicable; NIHSS, National Institutes of Health Stroke Scale; European Quality Five-Dimension Five-Level Self-Report Questionnaire (EQ-5D-5L); ICH, intracerebral hemorrhage.

^a^ Values were adjusted for age, baseline NIHSS score, baseline ASPECTS, occlusion site, and time from last known well to randomization, as prespecified in the protocol and statistical analysis plan.

^b^ The β coefficient was estimated from a linear regression model.

^c^ Recanalization on follow-up CTA or MRA within 48 hours data was not available for 32 and 40 patients in placebo group and tirofiban group, respectively.

^d^ Symptomatic ICH within 48 hours data was not available for 2 patient in placebo group.

^e^ Any radiologic ICH within 48 hours data was not available for 2 patient in placebo group.

## Table S4. Adverse events and complication according to treatment group in participants with large vessel occlusion stroke

|  | **Placebo group (n=333)** | **Tirofiban group (n=319)** | **Difference**  **(95% CI)** | **Risk Ratio**  **(95% CI)** | **P value** |
| --- | --- | --- | --- | --- | --- |
| **Severe adverse events** |  |  |  |  |  |
| Respiratory failure (%) | 47 (14.1) | 55 (17.2) | 0.03 (-0.03 to 0.09) | 1.27 (0.83-1.94) | 0.27 |
| Circulatory failure (%) | 34 (10.2) | 37 (11.6) | 0.02 (-0.03 to 0.07) | 1.15 (0.71-1.89) | 0.57 |
| Venous thrombosis (%) | 46 (13.8) | 29 (9.1) | 0.05 (-0.002 to 0.097) | 0.62 (0.38-1.02) | 0.06 |
| Decompressive craniectomy (%) | 7 (2.1) | 10 (3.1) | 0.001 (-0.02 to 0.04) | 1.51 (0.57-4.01) | 0.41 |
| Herniation (%) | 23 (6.9) | 26 (8.2) | 0.01 (-0.03 to 0.05) | 1.20 (0.67-2.14) | 0.55 |
| **Procedure associated complication** |  |  |  |  |  |
| Artery perforation (%) | 6 (1.8) | 9 (2.8) | 0.01 (-0.01 to 0.04) | 1.58 (0.56-4.50) | 0.39 |
| Artery dissection (%) | 10 (3.0) | 7 (2.2) | 0.01 (-0.02 to 0.04) | 0.73 (0.27-1.93) | 0.52 |
| Clot migration (%) | 54 (16.2) | 44 (13.8) | 0.02 (-0.03 to 0.08) | 0.83 (0.54-1.27) | 0.39 |
| Distal embolization (%) | 68 (20.4) | 65 (20.4) | 0.001 (-0.061 to 0.063) | 1.00 (0.68-1.46) | 0.99 |

## Table S5. Baseline Characteristics and workflow measures of the Patients and Features in sensitivity analysis

| **Characteristic** | **Overall (n=652)** | **Placebo group (n=302)** | **Tirofiban group (n=350)** | **P value** |
| --- | --- | --- | --- | --- |
| **Demographic characteristics** |  |  |  |  |
| Age, median (IQR) | 68 (58-75) | 69 (59-76) | 67 (58-74) | 0.11 |
| Sex, no. (%) |  |  |  | 0.41 |
| Women | 278 (42.6) | 134 (44.4) | 144 (41.1) |  |
| Men | 374 (57.4) | 168 (55.6) | 206 (58.9) |  |
| **Medical history, no. (%) ^a^** |  |  |  |  |
| Hypertension | 371 (56.9) | 167 (55.3) | 204 (58.3) | 0.44 |
| Diabetes | 152 (23.3) | 66 (21.9) | 86 (24.6) | 0.41 |
| Hyperlipidemia | 92 (14.1) | 40 (13.2) | 52 (14.9) | 0.56 |
| Atrial fibrillation | 232 (35.6) | 111 (36.8) | 121 (34.6) | 0.56 |
| Ischemic stroke | 112 (17.2) | 55 (18.2) | 57 (16.3) | 0.52 |
| Smoking (Current or Past) | 147 (22.5) | 69 (22.8) | 78 (22.3) | 0.86 |
| Prestroke modified Rankin scale score | | |  | 0.21 |
| 0 | 592 (90.8) | 269 (89.1) | 323 (92.3) |  |
| 1 | 42 (6.4) | 25 (8.3) | 17 (4.9) |  |
| 2 | 16 (2.5) | 7 (2.3) | 9 (2.6) |  |
| 3 | 1 (0.2) | 1 (0.3) | 0 (0.0) |  |
| 4 | 1 (0.2) | 0 (0.0) | 1 (0.3) |  |
| **Stroke etiology, no. (%)** | |  |  | 0.29 |
| Large artery atherosclerosis | 287 (44.0) | 126 (41.7) | 161 (46.0) |  |
| Cardioembolism | 299 (45.9) | 150 (49.7) | 149 (42.6) |  |
| Unknown | 48 (7.4) | 19 (6.3) | 29 (8.3) |  |
| Other | 18 (2.8) | 7 (2.3) | 11 (3.1) |  |
| **Clinical characteristics** |  |  |  |  |
| Baseline NIHSS score, median (IQR) | 16 (12-19) | 16 (12-20) | 16 (12-19) | 0.16 |
| Baseline Systolic blood pressure, median (IQR), mmHg | 145 (130-160) | 144 (129-159) | 146 (130-160) | 0.42 |
| Baseline Diastolic blood pressure, median (IQR), mmHg | 84 (76-94) | 84 (76-93) | 83 (75-94) | 0.97 |
| Baseline Serum glucose, median (IQR), mmol/L **^b^** | 6.9 (5.8-8.7) | 6.9 (5.8-8.7) | 6.9 (5.7-8.7) | 0.80 |
| **Imaging characteristics, no. (%)** | | |  | 0.17 |
| NCCT | 271 (41.6) | 137 (45.4) | 134 (38.3) |  |
| CTP | 288 (44.2) | 123 (40.7) | 165 (47.1) |  |
| MRI | 93 (14.3) | 42 (13.9) | 51 (14.6) |  |
| Baseline ASPECTS, median (IQR) | 8 (7-9) | 8 (7-9) | 8 (7-9) | 0.64 |
| Occlusion site | |  |  | 0.16 |
| ICA intracranial | 130 (19.9) | 63 (20.9) | 67 (19.1) |  |
| Middle cerebral artery |  |  |  |  |
| M1 segment | 424 (65.0) | 186 (61.6) | 238 (68.0) |  |
| M2 segment | 98 (15.0) | 53 (17.5) | 45 (12.9) |  |
| **Collateral status, no. (%) ^c^** | | |  | 0.18 |
| ASITN/SIR grade 0 | 54 (8.3) | 31 (10.3) | 23 (6.6) |  |
| ASITN/SIR grade 1 | 151 (23.2) | 76 (25.2) | 75 (21.5) |  |
| ASITN/SIR grade 2 | 261 (40.1) | 115 (38.1) | 146 (41.8) |  |
| ASITN/SIR grade 3 | 179 (27.5) | 76 (25.2) | 103 (29.5) |  |
| ASITN/SIR grade 4 | 6 (0.9) | 4 (1.3) | 2 (0.6) |  |
| **Tandem lesion, no. (%)** | |  |  | 0.79 |
| No | 617 (94.6) | 287 (95.0) | 330 (94.3) |  |
| Severe stenosis of extracranial segment (>=70%) | 25 (3.8) | 10 (3.3) | 15 (4.3) |  |
| Extracranial occlusion | 10 (1.5) | 5 (1.7) | 5 (1.4) |  |
| Total passes, median (IQR) | 2 (1-2) | 2 (1-2) | 1 (1-2) | 0.59 |
| First pass effect, no. (%) | 178 (27.3) | 81 (26.8) | 97 (27.7) | 0.80 |
| Stent thrombectomy only, no. (%) | 93 (14.3) | 50 (16.6) | 43 (12.3) | 0.12 |
| Aspiration only, no. (%) | 126 (19.3) | 58 (19.2) | 68 (19.4) | 0.94 |
| Salvage therapy, no. (%) | 129 (19.8) | 53 (17.5) | 76 (21.7) | 0.18 |
| SWIM only, no. (%) | 119 (18.3) | 55 (18.2) | 64 (18.3) | 0.98 |
| **Anesthesia, no. (%)** | |  |  | 0.96 |
| General | 226 (34.7) | 105 (34.8) | 121 (34.6) |  |
| Local | 426 (65.3) | 197 (65.2) | 229 (65.4) |  |
| Onset to randomization, median (IQR), min | 386 (283-619) | 359 (270-590) | 400 (292-631) | 0.08 |
| Door to puncture, median (IQR), min | 105 (78-144) | 99 (79-139) | 110 (78-150) | 0.04 |
| Puncture to recanalization, median (IQR), min | 70 (40-108) | 66 (40-105) | 72 (42-110) | 0.54 |

Abbreviations: IQR, interquartile range; NCCT, non-contrast computed tomography; CTP, computed tomography perfusion; MRI, magnetic resonance imaging; ASPECTS, Alberta Stroke Program Early CT Score; ASITN/SIR, American Society of Intervention and Therapeutic Neuroradiology/Society of Interventional Radiology; NIHSS, National Institutes of Health Stroke Scale; ICA, internal carotid artery.

^a^ Patient self-report or family report.

^b^ Serum glucose data was not available for 18 and 23 patients in placebo group and tirofiban group, respectively.

^c^ ASITN/SIR by DSA data was not available for 1 patient in tirofiban group.

##

## Table S6. Efficacy Outcomes and Primary Safety Outcomes in sensitivity analysis

| **Characteristic** | **Placebo group (n=302)** | **Tirofiban group (n=350)** | **Unadjusted difference (95% CI)** | **Unadjusted OR (95%CI)** | **Adjusted OR (95%CI) ^a^** | **P value** |
| --- | --- | --- | --- | --- | --- | --- |
| **Primary efficacy outcome** |  |  |  |  |  |  |
| mRS score at 90 days, median (IQR) | 3 (1-4) | 3 (1-4) | 0 (0 to 0) | 0.97 (0.74-1.26) | 0.88 (0.67-1.16) | 0.84 |
| **Secondary efficacy outcomes** |  |  |  |  |  |  |
| Excellent outcomes at 90 days (%) | 101 (33.4) | 116 (33.1) | 0.002 (-0.07 to 0.07) | 0.99 (0.71-1.37) | 0.89 (0.63-1.27) | 0.52 |
| Independent outcomes at 90 days (%) | 140 (46.4) | 168 (48.0) | 0.02 (-0.06 to 0.09) | 1.07 (0.79-1.45) | 0.99 (0.71-1.38) | 0.97 |
| Favorable outcomes at 90 days (%) | 191 (63.2) | 217 (62.0) | 0.01 (-0.06 to 0.08) | 0.95 (0.69-1.30) | 0.85 (0.60-1.20) | 0.34 |
| NIHSS change from baseline to 24 hours, median (IQR) **^b^** | -2 (-6 to 2) | -2 (-7 to 2) | 0 (-1 to 2) | 0.64 (-0.77 to 2.05) | 0.59 (-0.80 to 1.98) | 0.41 |
| NIHSS change from baseline to 5~7 days or early discharge, median (IQR) **^b^** | -5.5 (-11 to 1) | -4 (-10 to 0) | 1 (-1 to 2) | 1.16 (-0.67 to 2.98) | 1.11 (-0.68 to 2.89) | 0.22 |
| EQ-5D-5L score at 90d, median (IQR) **^b^** | 0.7 (0.2 to 1.0) | 0.7 (0.2 to 1.0) | 0 (0 to 0) | -0.01 (-0.07 to 0.05) | -0.03 (-0.08 to 0.03) | 0.33 |
| **Secondary technical efficacy outcomes** |  |  |  |  |  |  |
| Substantial reperfusion on initial DSA prior to EVT (%) | 1 (0.3) | 0 (0.0) | 0.003 (-0.01 to 0.02) | NA | NA | NA |
| Substantial reperfusion at final angiogram EVT (%) | 274 (90.7) | 320 (91.4) | 0.01 (-0.04 to 0.05) | 1.09 (0.64-1.87) | 1.07 (0.62-1.86) | 0.80 |
| Recanalization on follow-up CTA or MRA within 48 hours (%) **^c^** | 204 (91.1) | 215 (84.0) | 0.06 (-0.01 to 0.13) | 0.51 (0.29-0.91) | 0.54 (0.30-0.96) | 0.04 |
| **Primary safety outcomes** |  |  |  |  |  |  |
| Symptomatic ICH within 48 hours (%) **^d^** | 18 (6.0) | 35 (10.0) | 0.04 (-0.002 to 0.08) | 1.76 (0.97-3.18) | 1.90 (1.04-3.48) | 0.04 |
| Any radiologic ICH within 48 hours (%) **^e^** | 87 (28.8) | 119 (34.1) | 0.05 (-0.02 to 0.12) | 1.28 (0.92-1.78) | 1.34 (0.95-1.89) | 0.09 |
| Death at 90 days (%) | 46 (15.2) | 71 (20.3) | 0.05 (-0.01 to 0.11) | 1.42 (0.94-2.13) | 1.58 (1.03-2.43) | 0.04 |

CIs, confidence intervals; CTA, computed tomography angiography; DSA, digital subtraction angiography; IQR, interquartile range; MRA, magnetic resonance angiography; NA, not applicable; NIHSS, National Institutes of Health Stroke Scale; European Quality Five-Dimension Five-Level Self-Report Questionnaire (EQ-5D-5L).

^a^ Values were adjusted for age, baseline NIHSS score, baseline ASPECTS, occlusion site, and time from last known well to randomization, as prespecified in the protocol and statistical analysis plan.

^b^ The β coefficient was estimated from a linear regression model.

^c^ Recanalization on follow-up CTA or MRA within 48 hours data was not available for 78 and 94 patients in placebo group and tirofiban group, respectively.

^d^ Symptomatic ICH within 48 hours data was not available for 1 patient in tirofiban group.

^e^ Any radiologic ICH within 48 hours data was not available for 1 patient in tirofiban group.

## Table S7. Adverse events and complications in sensitivity analysis

|  | **Placebo group (n=302)** | **Tirofiban group (n=350)** | **Difference**  **(95% CI)** | **Risk Ratio**  **(95% CI)** | **P value** |
| --- | --- | --- | --- | --- | --- |
| **Severe adverse events** |  |  |  |  |  |
| Respiratory failure (%) | 40 (13.2) | 62 (17.7) | 0.05 (-0.01 to 0.10) | 1.41 (0.92-2.17) | 0.12 |
| Circulatory failure (%) | 27 (8.9) | 44 (12.6) | 0.03 (-0.01 to 0.08) | 1.47 (0.88-2.43) | 0.14 |
| Venous thrombosis (%) | 40 (13.2) | 35 (10.0) | 0.03 (-0.02 to 0.08) | 0.73 (0.45-1.18) | 0.20 |
| Decompressive craniectomy (%) | 5 (1.7) | 12 (3.4) | 0.02 (-0.01 to 0.04) | 2.11 (0.73-6.06) | 0.16 |
| Herniation (%) | 19 (6.3) | 30 (8.6) | 0.02 (-0.02 to 0.06) | 1.40 (0.77-2.54) | 0.27 |
| **Procedure associated complication** |  |  |  |  |  |
| Artery perforation (%) | 6 (2.0) | 9 (2.6) | 0.01 (-0.02 to 0.03) | 1.30 (0.46-3.70) | 0.62 |
| Artery dissection (%) | 5 (1.7) | 12 (3.4) | 0.02 (-0.01 to 0.04) | 2.11 (0.73-6.06) | 0.16 |
| Clot migration (%) | 49 (16.2) | 49 (14.0) | 0.02 (-0.03 to 0.08) | 0.84 (0.55-1.29) | 0.43 |
| Distal embolization (%) | 65 (21.5) | 68 (19.4) | 0.02 (-0.04 to 0.08) | 0.88 (0.60-1.29) | 0.51 |

## Table S8. Baseline Characteristics and workflow measures of the Patients in large artery atherosclerosis stratum

| Characteristic | Placebo group (n=156) | Tirofiban group (n=131) | P value |
| --- | --- | --- | --- |
| Demographic characteristics |  |  |  |
| Age, median (IQR) | 65 (56-73) | 65 (57-722) | 0.71 |
| Sex, no. (%) |  |  | 0.18 |
| Women | 41 (26.3) | 45 (34.4) |  |
| Men | 115 (73.7) | 86 (65.6) |  |
| Medical history, no. (%) ^a^ |  |  |  |
| Hypertension | 112 (71.8) | 90 (68.7) | 0.57 |
| Diabetes | 46 (29.5) | 42 (32.1) | 0.64 |
| Hyperlipidemia | 28 (17.9) | 28 (21.4) | 0.47 |
| Atrial fibrillation | 14 (9.0) | 13 (9.9) | 0.78 |
| Ischemic stroke | 27 (17.3) | 20 (15.3) | 0.64 |
| Smoking (Current or Past) | 60 (38.5) | 38 (29.0) | 0.09 |
| Prestroke modified Rankin scale score (fisher) |  |  | 0.08 |
| 0 | 143 (91.7) | 125 (95.4) |  |
| 1 | 12 (7.7) | 3 (2.3) |  |
| 2 | 1 (0.6) | 2 (1.5) |  |
| 4 | 0 (0.0) | 1 (0.8) |  |
| Clinical characteristics |  |  |  |
| Baseline NIHSS score, median (IQR) | 14 (10-19) | 15 (11-19) | 0.38 |
| Baseline Systolic blood pressure, median (IQR), mmHg | 148 (132-164) | 150 (138-169) | 0.22 |
| Baseline Diastolic blood pressure, median (IQR), mmHg | 85 (78-98) | 85 (78-95) | 0.99 |
| Baseline Serum glucose, median (IQR), mmol/L ^b^ | 7.0 (5.7-10.0) | 7.0 (5.9-10.2) | 0.81 |
| Baseline ASPECTS, median (IQR) | 8 (6-9) | 8 (7-9) | 0.85 |
| Occlusion site, no. (%) |  |  | 0.06 |
| ICA intracranial | 21 (13.5) | 24 (18.3) |  |
| Middle cerebral artery |  |  |  |
| M1 segment | 108 (69.2) | 96 (73.3) |  |
| M2 segment | 27 (17.3) | 11 (8.4) |  |
| Collateral status, no. (%) ^c^ |  |  | 0.67 |
| ASITN/SIR grade 0 | 11 (7.1) | 8 (6.1) |  |
| ASITN/SIR grade 1 | 28 (17.9) | 16 (12.2) |  |
| ASITN/SIR grade 2 | 64 (41.0) | 55 (42.0) |  |
| ASITN/SIR grade 3 | 51 (32.7) | 50 (38.2) |  |
| ASITN/SIR grade 4 | 2 (1.3) | 2 (1.5) |  |
| Tandem lesion, no. (%) |  |  | 0.66 |
| No | 145 (92.9) | 119 (90.8) |  |
| Severe stenosis of extracranial segment (>=70%) | 7 (4.5) | 9 (6.9) |  |
| Extracranial occlusion | 4 (2.6) | 3 (2.3) |  |
| Total passes, median (IQR) | 1 (1-3) | 1 (1-2) | 0.004 |
| First pass effect, no. (%) | 21 (13.5) | 28 (21.4) | 0.08 |
| Stent thrombectomy only, no. (%) | 17 (10.9) | 10 (7.6) | 0.35 |
| Aspiration only, no. (%) | 12 (7.7) | 9 (6.9) | 0.79 |
| Salvage therapy, no. (%) | 67 (42.9) | 50 (38.2) | 0.41 |
| SWIM only, no. (%) | 17 (10.9) | 22 (16.8) | 0.15 |
| Anesthesia, no. (%) |  |  | 0.38 |
| General | 61 (39.1) | 58 (44.3) |  |
| Local | 95 (60.9) | 73 (55.7) |  |
| Onset to randomization, median (IQR), min | 384 (246-648) | 386 (275-666) | 0.67 |
| Door to puncture, median (IQR), min | 117 (87-162) | 125 (89-168) | 0.42 |
| Puncture to recanalization, median (IQR), min | 90 (55-133) | 83 (47-135) | 0.50 |

Abbreviations: IQR, interquartile range; EVT, endovascular therapy; NCCT, non-contrast computed tomography; ASPECTS, Alberta Stroke Program Early CT Score; ASITN/SIR, American Society of Intervention and Therapeutic Neuroradiology/Society of Interventional Radiology; NIHSS, National Institutes of Health Stroke Scale; ICA, internal carotid artery.

^a^ Patient self-report or family report.

^b^ Serum glucose data was not available for 10 and 8 patients in placebo group and tirofiban group, respectively.

## Table S9. Analysis of clinical and safety outcomes in large artery atherosclerosis stratum

| **Characteristic** | **Placebo group (n=156)** | **Tirofiban group (n=131)** | **Unadjusted difference (95% CI)** | **Unadjusted OR (95%CI)** | **Adjusted OR (95%CI)** ^a^ | **P value** |
| --- | --- | --- | --- | --- | --- | --- |
| **Primary efficacy outcome** |  |  |  |  |  |  |
| mRS score at 90 days, median (IQR) | 3 (2-5) | 3 (1-4) | 0 (-1 to 0) | 1.48 (0.98-2.22) | 1.74 (1.14-2.65) | 0.01 |
| **Secondary efficacy outcomes** |  |  |  |  |  |  |
| Excellent outcomes at 90 days (%) | 38 (24.4) | 40 (30.5) | 0.06 (-0.04 to 0.16) | 1.37 (0.81-2.30) | 1.61 (0.91-2.86) | 0.10 |
| Independent outcomes at 90 days (%) | 55 (35.3) | 63 (48.1) | 0.13 (0.01 to 0.24) | 1.70 (1.06-2.74) | 2.04 (1.21-3.44) | 0.008 |
| Favorable outcomes at 90 days (%) | 86 (55.1) | 84 (64.1) | 0.08 (-0.03 to 0.20) | 1.46 (0.90-2.34) | 1.66 (0.98-2.79) | 0.06 |
| NIHSS change from baseline to 24 hours, median (IQR) ^b^ | -1.0 (-4.0 to 4.0) | -1.0 (-6.0 to 3.0) | 0 (-1 to 1) | 0.87 (0.11-6.74) | 0.98 (0.13-7.47) | 0.66 |
| NIHSS change from baseline to 5~7 days or early discharge, median (IQR) ^b^ | -2.0 (-7.0 to 4.0) | -4.0 (-10.0 to 1.0) | -2 (-4 to 0) | 0.21 (0.01-3.22) | 0.20 (0.01-2.94) | 0.07 |
| EQ-5D-5L score at 90d, median (IQR) ^b^ | 0.5 (0.1 to 0.9) | 0.7 (0.2 to 1.0) | 0.05 (0 to 0.2) | 0.09 (-0.00-0.18) | 1.11(1.02-1.21) | 0.06 |
| **Secondary technical efficacy outcomes** |  |  |  |  |  |  |
| Substantial reperfusion on initial DSA prior to EVT (%) | 1 (0.6) | 0 (0.0) | 0.01 (-0.02 to 0.04) | NA | NA | NA |
| Substantial reperfusion at final angiogram EVT (%) | 135 (86.5) | 113 (86.3) | 0.002 (-0.08 to 0.08) | 0.98 (0.50-1.92) | 0.98 (0.49-1.96) | 0.94 |
| Recanalization on follow-up CTA or MRA within 48 hours (%) ^c^ | 91 (77.8) | 72 (75.0) | 0.04 (-0.08 to 0.15) | 0.86 (0.45-1.62) | 0.84 (0.43-1.63) | 0.61 |
| Reocclusion within 48 hours (%) ^d^ | 21 (19.3) | 21 (23.1) | 0.04 (-0.08 to 0.16) | 1.26 (0.64-2.49) | 1.25 (0.62-2.54) | 0.53 |
| **Primary safety outcomes** |  |  |  |  |  |  |
| Symptomatic ICH within 48 hours (%) ^e^ | 11 (7.1) | 12 (9.2) | 0.02 (-0.04 to 0.09) | 1.34 (0.57-3.15) | 1.40 (0.58-3.36) | 0.45 |
| Any radiologic ICH within 48 hours (%) ^f^ | 37 (23.7) | 41 (31.5) | 0.07 (-0.03 to 0.18) | 1.48 (0.88-2.50) | 1.60 (0.92-2.76) | 0.09 |
| Death at 90 days (%) | 35 (22.4) | 21 (16.0) | 0.06 (-0.03 to 0.15) | 0.66 (0.36-1.20) | 0.58 (0.30-1.11) | 0.10 |

CIs, confidence intervals; CTA, computed tomography angiography; DSA, digital subtraction angiography; IQR, interquartile range; MRA, magnetic resonance angiography; NA, not applicable; NIHSS, National Institutes of Health Stroke Scale; European Quality Five-Dimension Five-Level Self-Report Questionnaire (EQ-5D-5L).

^a^ Values were adjusted for age, baseline NIHSS score, baseline ASPECTS, occlusion site, and time from last known well to randomization, as prespecified in the protocol and statistical analysis plan.

^b^ The β coefficient was estimated from a linear regression model.

^c^ Recanalization on follow-up CTA or MRA within 48 hours data was not available for 39 and 35 patients in placebo group and tirofiban group, respectively.

^d^ Reocclusion within 48 hours data was not available for 47 and 40 patients in placebo group and tirofiban group, respectively.

^e^ Symptomatic ICH within 48 hours data was not available for 1 patient in tirofiban group.

^f^ Any radiologic ICH within 48 hours data was not available for 1 patient in tirofiban group.

## Table S10. Baseline Characteristics and workflow measures of the Patients in cardioembolic stroke stratum

| Characteristic | Placebo group (n=151) | Tirofiban group (n=148) | P value |
| --- | --- | --- | --- |
| Demographic characteristics |  |  |  |
| Age, median (IQR) | 72 (62-77) | 72 (65-77) | 0.66 |
| Sex, no. (%) |  |  | 0.54 |
| Women | 87 (57.6) | 80 (54.1) |  |
| Men | 64 (42.4) | 68 (45.9) |  |
| Medical history, no. (%) ^a^ |  |  |  |
| Hypertension | 66 (43.7) | 73 (49.3) | 0.33 |
| Diabetes | 22 (14.6) | 31 (20.9) | 0.15 |
| Hyperlipidemia | 15 (9.9) | 17 (11.5) | 0.66 |
| Atrial fibrillation | 97 (64.2) | 97 (65.5) | 0.81 |
| Ischemic stroke | 29 (19.2) | 32 (21.6) | 0.60 |
| Smoking (Current or Past) | 14 (9.3) | 22 (14.9) | 0.14 |
| Prestroke modified Rankin scale score |  |  | 0.93 |
| 0 | 131 (86.8) | 131 (88.5) |  |
| 1 | 14 (9.3) | 12 (8.1) |  |
| 2 | 6 (4.0) | 5 (3.4) |  |
| Clinical characteristics |  |  |  |
| Baseline NIHSS score, median (IQR) | 17 (14-21) | 16 (12-19) | 0.05 |
| Baseline Systolic blood pressure, median (IQR), mmHg | 142 (126-156) | 142 (124-158) | 0.78 |
| Baseline Diastolic blood pressure, median (IQR), mmHg | 81 (74-92) | 80 (75-90) | 0.69 |
| Baseline Serum glucose, median (IQR), mmol/L ^b^ | 6.9 (5.8-8.4) | 6.9 (5.7-8.6) | 0.90 |
| Baseline ASPECTS, median (IQR) | 8 (7-9) | 8 (7-9) | 0.41 |
| Occlusion site, no. (%) |  |  | 0.29 |
| ICA intracranial | 41 (27.2) | 29 (19.6) |  |
| Middle cerebral artery |  |  |  |
| M1 segment | 86 (57.0) | 95 (64.2) |  |
| M2 segment | 24 (15.9) | 24 (16.2) |  |
| Collateral status, no. (%) ^c^ |  |  | 0.33 |
| ASITN/SIR grade 0 | 19 (12.6) | 10 (6.8) |  |
| ASITN/SIR grade 1 | 42 (27.8) | 44 (29.9) |  |
| ASITN/SIR grade 2 | 64 (42.4) | 62 (42.2) |  |
| ASITN/SIR grade 3 | 25 (16.6) | 31 (21.1) |  |
| ASITN/SIR grade 4 | 1 (0.7) | 0 (0.0) |  |
| Tandem lesion, no. (%) |  |  | 0.62 |
| No | 150 (99.3) | 146 (98.6) |  |
| Severe stenosis of extracranial segment (>=70%) | 1 (0.7) | 2 (1.4) |  |
| Total passes, median (IQR) | 2 (1-3) | 2 (1-2) | 0.30 |
| First pass effect, no. (%) | 49 (32.5) | 57 (38.5) | 0.27 |
| Stent thrombectomy only, no. (%) | 29 (19.2) | 22 (14.9) | 0.32 |
| Aspiration only, no. (%) | 43 (28.5) | 51 (34.5) | 0.27 |
| Salvage therapy, no. (%) (fisher) | 4 (2.6) | 2 (1.4) | 0.68 |
| SWIM only, no. (%) | 33 (21.9) | 30 (20.3) | 0.74 |
| Anesthesia, no. (%) |  |  | 0.98 |
| General | 40 (26.5) | 39 (26.4) |  |
| Local | 111 (73.5) | 109 (73.6) |  |
| Onset to randomization, median (IQR), min | 258 (179-345) | 288 (194-357) | 0.23 |
| Door to puncture, median (IQR), min | 90 (65-130) | 100 (70-130) | 0.21 |
| Puncture to recanalization, median (IQR), min | 58 (35-89) | 60 (34-90) | 0.65 |

Abbreviations: IQR, interquartile range; EVT, endovascular therapy; NCCT, non-contrast computed tomography; ASPECTS, Alberta Stroke Program Early CT Score; ASITN/SIR, American Society of Intervention and Therapeutic Neuroradiology/Society of Interventional Radiology; NIHSS, National Institutes of Health Stroke Scale; ICA, internal carotid artery.

^a^ Patient self-report or family report.

^b^ Serum glucose data was not available for 6 and 10 patients in placebo group and tirofiban group, respectively.

^c^ ASITN/SIR by DSA data was not available for 1 patient in tirofiban group.

## Table S11. Analysis of clinical and safety outcomes in cardioembolic stroke stratum

| **Characteristic** | **Placebo group (n=151)** | **Tirofiban group (n=148)** | **Unadjusted difference (95% CI)** | **Unadjusted OR (95%CI)** | **Adjusted OR (95%CI)** ^a^ | **P value** |
| --- | --- | --- | --- | --- | --- | --- |
| **Primary efficacy outcome** |  |  |  |  |  |  |
| mRS score at 90 days, median (IQR) | 2 (1-4) | 2 (1-5) | 0 (0 to 0) | 0.96 (0.64-1.43) | 0.79 (0.53-1.20) | 0.27 |
| **Secondary efficacy outcomes** |  |  |  |  |  |  |
| Excellent outcomes at 90 days (%) | 57 (37.7) | 59 (39.9) | 0.02 (-0.09 to 0.13) | 1.09 (0.69-1.74) | 84 (0.49-1.45) | 0.54 |
| Independent outcomes at 90 days (%) | 81 (53.6) | 78 (52.7) | 0.01 (-0.10 to 0.12) | 0.96 (0.61-1.52) | 0.82 (0.48-1.38) | 0.45 |
| Favorable outcomes at 90 days (%) | 100 (66.2) | 96 (64.9) | 0.01 (-0.09 to 0.12) | 0.94 (0.58-1.52) | 0.84 (0.48-1.45) | 0.53 |
| NIHSS change from baseline to 24 hours, median (IQR) ^b^ | -4.0 (-9.0 to 0.0) | -4.0 (-9.0 to 1.0) | 0 (-2 to 2) | 0.87 (-1.21 to 2.95) | 0.95 (-1.12 to 3.02) | 0.37 |
| NIHSS change from baseline to 5~7 days or early discharge, median (IQR) ^b^ | -8.0 (-13.0 to -2.0) | -6.0 (-12.0 to 0.0) | 1 (0 to 3) | 2.07 (-0.56 to 4.70) | 2.08 (-0.52 to 4.69) | 0.12 |
| EQ-5D-5L score at 90d, median (IQR) ^b^ | 0.8 (0.2 to 1.0) | 0.8 (0.2 to 1.0) | 0.00 (-0.05 to 0.05) | -0.02 (-0.11 to 0.07) | -0.06 (-0.14 to-0.02) | 0.17 |
| **Secondary technical efficacy outcomes** |  |  |  |  |  |  |
| Substantial reperfusion on initial DSA prior to EVT (%) | 1 (0.6) | 0 (0.0) | 0.01 (-0.02 to 0.04) | NA | NA | NA |
| Substantial reperfusion at final angiogram EVT (%) | 143 (94.7) | 142 (95.9) | 0.01 (-0.04 to 0.07) | 0.98 (0.50-1.92) | 1.21 (0.38-3.80) | 0.75 |
| Recanalization on follow-up CTA or MRA within 48 hours (%) ^c^ | 110 (96.5) | 105 (96.3) | 0.05 (-0.05 to 0.15) | 0.96 (0.23-3.92) | 1.13 (0.26-5.03) | 0.87 |
| Reocclusion within 48 hours (%) ^d^ | 4 (3.6) | 4 (3.7) | 0.00 (-0.04 to 0.04) | 1.04 (0.25-4.26) | 0.88 (0.20-3.92) | 0.87 |
| **Primary safety outcomes** |  |  |  |  |  |  |
| Symptomatic ICH within 48 hours (%) | 7 (4.6) | 16 (10.8) | 0.06 (0.00 to 0.13) | 2.49 (1.00-6.25) | 3.27 (1.24-8.61) | 0.02 |
| Any radiologic ICH within 48 hours (%) | 46 (30.5) | 59 (39.9) | 0.09 (-0.01 to 0.20) | 1.51 (0.94-2.44) | 1.71 (1.04-2.83) | 0.04 |
| Death at 90 days (%) | 18 (11.9) | 31 (20.9) | 0.09 (-0.01 to 0.17) | 1.96 (1.04-3.68) | 2.32 (1.20-4.51) | 0.01 |

CIs, confidence intervals; CTA, computed tomography angiography; DSA, digital subtraction angiography; IQR, interquartile range; MRA, magnetic resonance angiography; NA, not applicable; NIHSS, National Institutes of Health Stroke Scale; European Quality Five-Dimension Five-Level Self-Report Questionnaire (EQ-5D-5L).

^a^ Values were adjusted for age, baseline NIHSS score, baseline ASPECTS, occlusion site, and time from last known well to randomization, as prespecified in the protocol and statistical analysis plan.

^b^ The β coefficient was estimated from a linear regression model.

^c^ Recanalization on follow-up CTA or MRA within 48 hours data was not available for 37 and 39 patients in placebo group and tirofiban group, respectively.

^d^ Reocclusion within 48 hours data was not available for 39 and 40 patients in placebo group and tirofiban group, respectively.
